# Spatial variability of soil texture fractal dimension and its application in sponge city construction

**DOI:** 10.1371/journal.pone.0357100

**Published:** 2026-09-21

**Authors:** Jianjun Wang, Zhaolin Lu, Jian Tang, Yun Li, Yonghui Li, Liansan Xu, Yanfeng Liu, Zhuqiang Jiang, Lichuan Luo, Baohong Li

**Affiliations:** 1 The Institute of Hydrogeologic and Engineering Geological of Wuhan, Hubei Province Geological Bureau, Wuhan, China; 2 Hubei Key Laboratory of Resource and Eco-Environment Geology, Wuhan, China; 3 School of Environmental Studies, China University of Geosciences, Wuhan, China; 4 Hubei Water Resources Technical Collage, Wuhan, China; Ural Federal University named after the first President of Russia B N Yeltsin: Ural'skij federal'nyj universitet imeni pervogo Prezidenta Rossii B N El'cina, RUSSIAN FEDERATION

## Abstract

Accurate characterization of spatial variability of soil textures and their relationships with hydraulic properties is essential for the planning, design, and construction of sponge cities. This study explored effective approaches for characterizing the spatial variability of soil textures and determining the soil hydraulic parameters in the sponge cities by using fractal theory and geostatistics. The results show that soil particle sizes in the study area are mainly distributed between 0.002 and 0.5 mm, with silt being the dominant fraction. The fractal dimensions (D values) of soil particle size distribution (PSD) ranged from 2.07 to 2.71, with an average of 2.50. The Semi-variogram models, including the spherical and Gaussian models were found suitable for soil D values at different depths. The spatial distribution of soil D values was illustrated in a three-dimensional model, and the fractal model of soil water characteristic curves was established to understand the soil infiltration and storage capacities. Overall, this research demonstrates that fractal theory provides an efficient approach to characterize the spatial distribution of soil PSD and establish the relationship between soil D values and hydraulic parameters, offering a scientific basis for planning and construction of sponge city not only in China, but also in similar regions worldwide.

## 1. Introduction

Global warming has intensified extreme climate events, leading to uneven spatial and temporal distributions of rainfall and water resources, along with a marked increase in the frequency and intensity of heavy precipitation events in recent decades [1–3]. Consequently, urban challenges such as stormwater flooding and water pollution have become increasingly severe, resulting in incidents of human casualties and substantial property losses. Statistical data indicate that nearly 200 cities and towns in China have experienced urban flooding [4,5]. To address urban waterlogging and enhance the efficient utilization of water resources, the concept of “sponge city”, also referred to the construction of low-impact development rainwater systems, has been proposed in China [6,7]. The primary purpose of sponge city construction is to create urban environments that naturally retain, infiltrate, and purify rainwater during rainfall events to promote its effective utilization [6–8]. Beyond mitigating water-related risks, sponge city development serves as a comprehensive strategy to address urban water security, water resource management, water quality, and ecological sustainability, while advancing the broader goal of ecological civilization development.

A critical foundation of sponge city planning lies in understanding the infiltration and storage capacities of urban soils. These properties are governed by soil hydraulic parameters, which significantly influence rainfall infiltration, soil water movement, and pollutant transport in the vadose zone. However, due to the complexity of soil texture and structure, these parameters exhibit strong heterogeneity and scale dependence. Traditional methods for determining soil hydraulic parameters are often time-consuming and labor-intensive, and are typically limited to shallow soil layers and small-scale field experiments [9–11]. This limitation hinders the comprehensive understanding of soil water behavior across different depths and land-use types, which is essential for the effective design of sponge city infrastructure.

As a typical porous medium, soil texture exhibits irregular shapes and self-similar structures, making it suitable for analysis using fractal theory [12–14]. Since Mandelbrot (1977) introduced fractal concepts into soil science, this approach has been widely applied to describe complex and irregular phenomena that classical geometric methods fail to capture [15]. Soil particle size distribution (PSD), a fundamental physical attribute reflecting soil composition and texture, has been shown to possess fractal parameters [16,17]. The fractal dimension derived from PSD is considered an effective indicator of soil texture and is closely related to hydraulic characteristics such as water retention and permeability [10,18–21]. These relationships offer a promising pathway for estimating soil hydraulic parameters in a more efficient and scalable manner.

Despite these advances, most existing studies have focused on the spatial variability of soil PSD or its correlation with general soil properties [14,22,23]. Limited attention has been paid to the vertical variability of fractal dimensions and their potential as indicators for hydraulic behavior in urban environments. Moreover, the application of fractal models in the context of sponge city construction remains underdeveloped, particularly in terms of evaluating how soil fractal characteristics at different depths influence the performance of sponge infrastructure.

This study addresses these gaps by systematically analyzing the spatial and vertical variability of soil fractal dimensions in the sponge city construction area of Wuhan. Unlike previous studies, we focus on the correlation between fractal dimensions and soil hydraulic parameters across multiple depths and urban land-use types. By integrating fractal modeling with soil water characteristic curves, we aim to provide a rapid and scalable method for estimating hydraulic parameters, thereby supporting more efficient and scientifically grounded sponge city planning. This approach not only enhances the mechanistic understanding of soil behavior in urban environments but also offers practical tools for optimizing sponge facility design in regions with complex soil structures.

The specific objectives of this study are to: (1) characterize the spatial and vertical distribution of soil PSD and fractal dimensions in the Wuhan sponge city area; (2) explore the relationship between fractal dimensions and soil hydraulic parameters; and (3) evaluate the applicability of fractal models for estimating soil water retention characteristics to inform sponge city design.

## 2. Materials and methods

### 2.1. Study area

**Fig 1:**
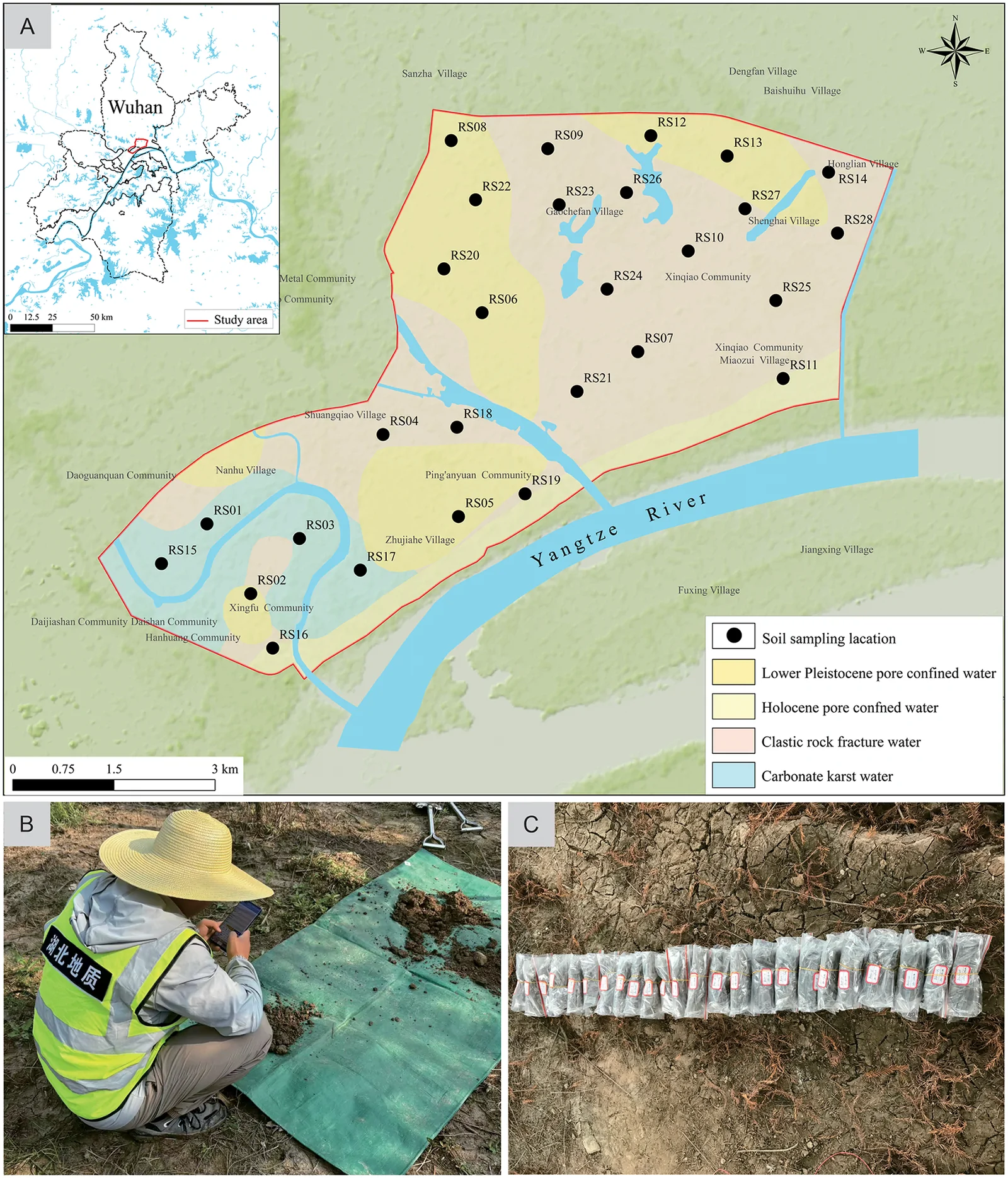
Schematic map of the study area, soil sampling locations and field sampling photographs. (A) Geographic location and hydrogeological zoning map of the Yangtze River New Town sponge city study area in Wuhan. (B) Field photograph showing on-site recording of sampling information by geological survey personnel during soil collection. (C) Photograph of labeled, sealed intact soil samples prepared for subsequent laboratory analysis.

The Wuhan sponge city construction area is situated in the initial section of the Yangtze River new town (YRNT), located between the north bank of the Yangze River and the confluence of the Fu and Sheshui Rivers, covering an area of approximately 50 km^2^ (Fig 1). The YRNT experiences a subtropical monsoon climate, characterized by hot, humid summers and cold winters. The mean annual air temperature ranges from 15.8 to17.5 °C, while the mean annual precipitation and evaporation are 1,252.2 mm and 1,447.9 mm, respectively. The study area is well-connected with water systems, being adjacent to the Yangtze River, Fu River, Sheshui River, and Daoshui River, as well as numerous lakes including Wu Lake and Hou Lake, resulting in a well-developed surface water network.

The geomorphology of the YRNT is predominantly a plain landscape, mainly composed of Grade I and Ⅱ terraces of the Yangtze River, featuring gentle and open terrain with elevations ranging from 16 to 30 meters above sea level (m asl). Quaternary deposits are widely throughout the area, including artificial fill, the Holocene Zoumaling Formation, the Late Pleistocene Xiashu Formation, the Middle Pleistocene Wangjiadian Formation, and the Early Pleistocene Yangluo Formation. The regional lithology primarily consists of clay, sandy clay, clayey sand, gravel-bearing clay, sandy soil, sand-gravel, and gravel layers, with an average thickness of approximately 30 meters [24].

Groundwater in the study area can be classified into three types: porous confined water in loose rocks, porous-fissure water in clastic rocks, and karst-fissure water in carbonate rocks. The primary aquifer is found in the Early Pleistocene Yangluo Formation. The upper Quaternary deposits consist of thick layers of clay and silty clay, while the lower aquifer exhibits confined conditions. Groundwater levels in the area ranges from 2.50 to 17.42 m in depth. Fig 1 illustrates the geographical setting, sampling campaign and field soil samples of this study. (A) The location map delineates the overall scope of the Yangtze River New Town study area (red boundary), with an inset small map marking the position of the research zone within Wuhan City. Four hydrogeological units are differentiated by distinct color coding: pale yellow for Lower Pleistocene porous confined aquifers, light beige for Holocene porous confined aquifers, light pink for clastic rock fracture aquifers, and light blue for carbonate karst aquifers; major villages, communities and the Yangtze River are also labelled for spatial reference. (B) The field photograph documents on-site soil sampling work. (C) Packaged intact soil core samples sealed in plastic bags with serial labels, prepared for subsequent laboratory physicochemical analyses.

### 2.2. Soil sampling and measurement

To investigate the lithological structure of the vadose zone, 28 soil sampling profiles were uniformly distributed across the study area (Fig 1A). Standard field sampling protocols were employed to collect soil samples, as illustrated by the technician processing samples on a tarp alongside systematically labeled sample bags prepared for subsequent analysis (Figs 1B and 1C). At each sampling point, soil cores were collected at 10 cm intervals to a maximum depth ranging from 150 to 240 cm. Concurrently, soil moisture content was measured at each interval using an MPKit406 device (ICT International).

The sampling locations were selected to represent diverse land-use types and geomorphological units within the study area, ensuring spatial coverage and representativeness [24]. All measurements were conducted in triplicate to ensure data reliability. The collected soil samples were air-dried, crushed, and passed through a 2 mm sieve in the laboratory prior to PSD analysis. Soil particle composition was determined using a Beckman Coulter LS 13 320 laser diffraction particle size analyzer, a widely used and highly precise instrument based on Fraunhofer diffraction and Mie scattering theories. This analyzer provides high resolution, excellent repeatability, accurate measurements, and rapid testing capabilities.

To prevent instrument clogging, air-dried soil samples were pre-sieved through a 0.9 mm standard sieve. The < 0.9 mm fraction was analyzed with a LS 13 320 laser diffraction analyzer, and the > 0.9 mm fraction was measured by dry sieve analysis. The full particle size distribution of each sample was calculated by weighting the mass proportion of the two fractions. According to the Chinese Standard for Engineering Classification of soil, the composition of particles is divided into clay (*d* < 0.005 mm), silt (0.005 mm ≤ *d* < 0.075 mm), and sand (0.075 mm ≤ *d* < 0.25 mm) [11]. Based on the particle test results, the dominant soil texture classes in the study area are silt, silty clay and clay.

### 2.3. Fractal theory

Mandelbrot (1977) first introduced the concept of fractals and fractal theory, providing a framework for analyzing complex, irregular phenomena that cannot be adequately described by classical geometric methods [15]. Studies have shown that soil exhibits fractal characteristics, with particles and pores of varying shapes and sizes, forming irregular geometric patterns [14,25].

The fractal dimension of soil PSD provides an efficient means of characterizing PSD and composition, reflecting the uniformity of soil texture [26]. It serves as a novel parameter that complements traditional particle-size statistical methods in soil analysis. Lower fractal dimensions indicate a looser soil structure and higher permeability, whereas higher fractal dimensions correspond to denser soil compaction and reduced permeability. The fractal dimension of the soil particle size was calculated using the volume-based fractal model proposed by Tyler and Wheatcraft (1992), which can be expressed as the follows:


$$\begin{array}{c} {\left(\frac{d_{i}}{d_{max}}\right)}^{3-D}=\frac{V\left(\delta <d_{i}\right)}{V_{\text{0}}} \end{array}$$ (1)

$$\begin{array}{c} \left(3-D\right)lg\frac{d_{i}}{d_{max}}=lg\frac{V\left(\delta <d_{i}\right)}{V_{\text{0}}} \end{array}$$ (2)

where *d*_*i*_ (*i* = 1, 2, 3,…, *n*) represents the diameter of soil particles, *d*_*max*_ is the maximum particle diameter, *D* is the fractal dimension, *δ* is the variable particle size, *V*(*δ* < *d*_*i*_) is the cumulative volume of particles with diameters smaller than *d*_*i*,_ and *V*_0_ is the total volume of the soil sample.

To calculated the fractal dimension (D value), $lg\frac{V\left(\delta <d_{i}\right)}{V_{\text{0}}}$ and $lg\frac{d_{i}}{d_{max}}$ were first derived from the measured particle sizes and their corresponding volume data. A plot of $lg\frac{V\left(\delta <d_{i}\right)}{V_{\text{0}}}$ versus $lg\frac{d_{i}}{d_{max}}$ was then constructed, and the slope (*k*) of the fitted line was obtained using least squares linear regression. The fractal dimension was subsequently calculated as D = 3 – *k*.

### 2.4. Geostatistical analysis

Geostatistics is a scientific discipline based on the theory of regionalized variables, employing semi-variograms as a primary tool to analyze natural phenomena that exhibit both random variation and structured spatial patterns [27,28]. As an effective method for investigating the spatial distribution and variability of soil properties, the semi-variogram describes both the structural and random components of regionalized variables.

Geostatistical techniques are commonly used to analyze the spatial variability of the fractal dimension of soil PSD [27,28]. The mathematical expression is as follows:


$$\begin{array}{c} \gamma \left(h\right)=\frac{\text{1}}{2N\left(h\right)}\sum_{i=1}^{N\left(h\right)}\left[Z\left(x_{i}\right)-Z\left(x_{i}+h\right)\right] \end{array}$$ (3)

where γ(*h*) represents the semi-variance at lag distance *h*, *N*(*h*) is the number of sample pairs separated by distance *h*, Z(*x*_*i*_) is the measured value at location *x*_*i*_, and Z(*x*_*i*_ + *h*) is the measured value at a location separated by distance *h*. In this study, the geostatistical software GS + 10.0 was used to analyze the semi-variogram function [29].

## 3. Results

### 3.1. Descriptive statistics of soil PSD

Soil particle size distribution (PSD) is a fundamental indicator for evaluating hydrodynamic conditions and understanding the variability of soil texture across spatial scales [30]. The PSD analysis results from 28 representative soil profiles are summarized in S1 Table and Table 1. The particle sizes in the study area are primarily concentrated between 0.002 mm and 0.5 mm, with silt being the dominant fraction.

**Table 1:** Descriptive statistical results of soil textural parameters.

Soil textural parameters	Min.	Max.	Mean	Standard deviation	Coefficient variation (%)
Sand (%)	0.20	71.50	8.96	6.33	71.21
Silt (%)	26.90	96.60	76.32	6.66	8.73
Clay (%)	1.6	26.9	14.80	2.99	20.27

The coefficient of variation (CV) was used to quantify soil property variability, classified as low (<10%), moderate (10–100%) and high (>100%) [31]. Silt had the highest mean content (76.32%) with low variability (CV = 8.73%), clay averaged 14.80% with moderate variability (CV = 20.27%), and sand had the lowest mean content (8.96%) but high variability (CV = 71.21%).

These results indicate that the soils in the study area are predominantly composed of fine particles, particularly silt and clay, which are known to influence water retention and infiltration behavior. The high variability in sand content suggests localized heterogeneity in soil texture, which may have implications for the spatial distribution of hydraulic properties in the vadose zone.

### 3.2 Fractal characteristics of soil PSD

Based on calculations from Equations (1) and (2), the statistical results of fractal dimensions (D values) of soil PSD at different depths in the study area are presented in Table 2. The D values range from 2.07 to 2.71, with an average of 2.50. For specific soil types, the fractal dimensions of silt, silty clay, and clay range from 2.07 to 2.49, 2.24 to 2.71, and 2.54 to 2.63 with average values of 2.39, 2.52 and 2.59, respectively. Compared with the 0–100 cm depth interval, D values at 100–240 cm show a narrower variation range, indicating higher homogeneity of soil particle composition and weaker spatial variability at greater depths. As shown in Table 2, the CV values of D for all soil profiles are below 10%, and drop to 1.1–2.3% at depths below 100 cm.

**Table 2:** Statistical results of fractal dimensions at different depths.

Depth (cm)	Soil texture	Min.	Max.	Mean	Skewness	Kurtosis	Coefficient variation
0-20	Silty clay	2.07	2.60	2.49	−1.85	5.28	3.07%
20-40	Silt, silty clay	2.10	2.66	2.51	−2.12	6.96	4.99%
40-60	Silty clay	2.09	2.71	2.51	1.25	3.69	2.78%
60-80	Silty clay	2.24	2.59	2.48	−1.50	2.74	3.69%
80-100	Silt, silty clay	2.27	2.60	2.50	−1.32	3.23	3.57%
100-120	Silty clay	2.46	2.59	2.53	0.34	−0.17	1.59%
120-140	Silt, silty clay	2.42	2.63	2.52	−0.39	−0.60	2.10%
140-160	Silt, silty clay	2.45	2.63	2.54	0.25	−0.63	2.30%
160-180	Silty clay, clay	2.43	2.60	2.52	−0.28	−0.95	1.99%
180-200	Silty clay	2.43	2.61	2.51	1.44	2.53	1.73%
200-220	Silt, silty clay	2.40	2.56	2.49	−0.13	−0.14	1.75%
220-240	Silty clay, clay	2.44	2.59	2.51	0.19	0.49	1.52%

**Fig 2:**
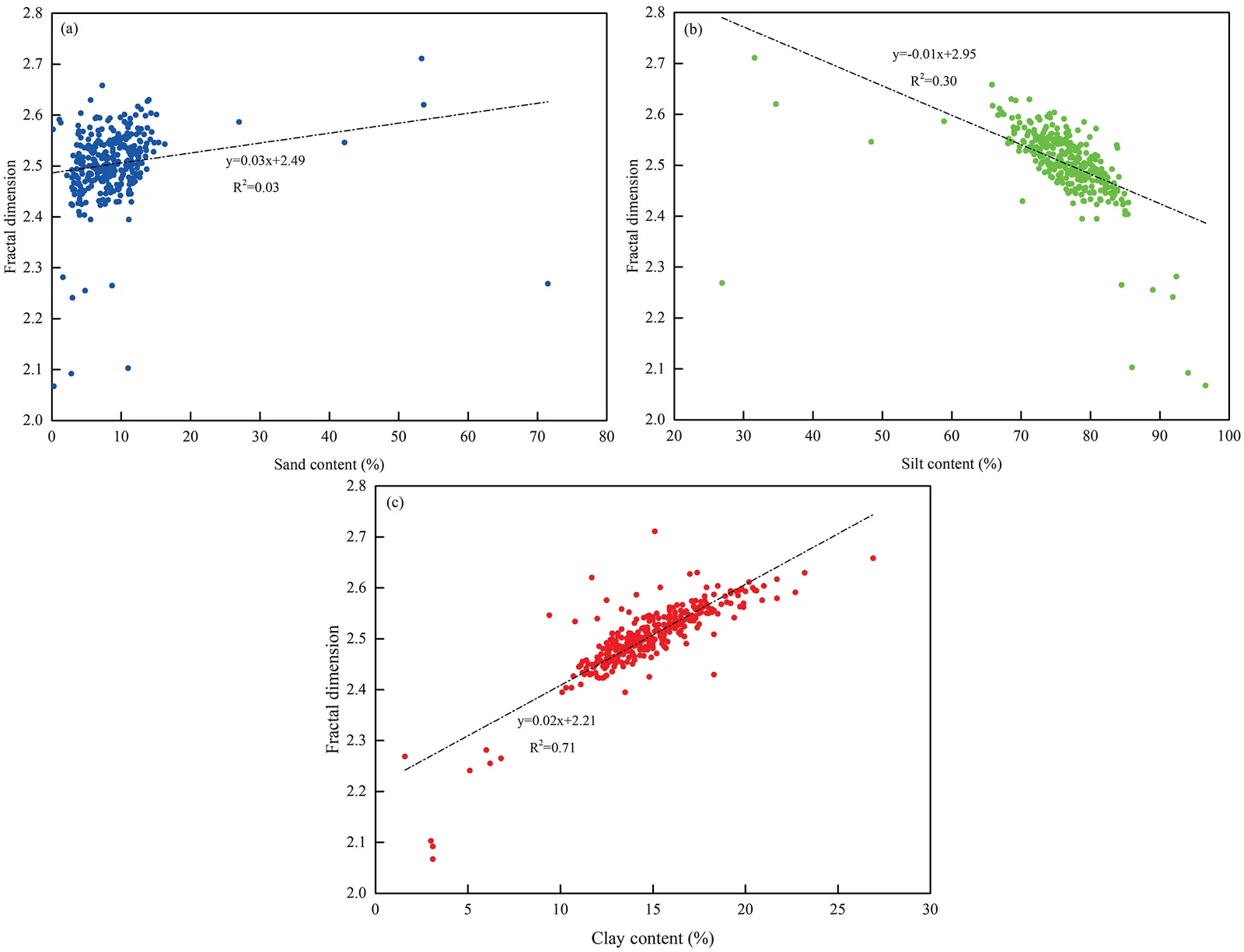
Scatter plot of fractal dimension vs. soil particle size compositions.

The relationships between sand, silt, and clay contents and the soil particle size fractal dimension in the study area are shown in Fig 2. Among the soil fractions, clay content exhibits the strongest correlation with the fractal dimension (R² > 0.7), followed by silt (R^2^ = 0.29), while sand shows the weakest correlation. These results indicate that clay has the greatest influence on soil fractal dimension in the study area, with the fractal dimension decreasing as soil texture becoming coarser.

### 3.3. Spatial variability of soil fractal dimension

To evaluate the spatial variability of soil PSD fractal dimensions, a normality test was first conducted using the Kolmogorov–Smirnov method. The results indicated that most depth intervals between 0 and 240 cm followed a normal distribution, except for the depths of 0–40 cm, 60–80 cm, 80–100 cm and 180–200 cm layers. These non-normal datasets were transformed using the Box–Cox method to meet the assumptions required for geostatistical analysis.

**Fig 3:**
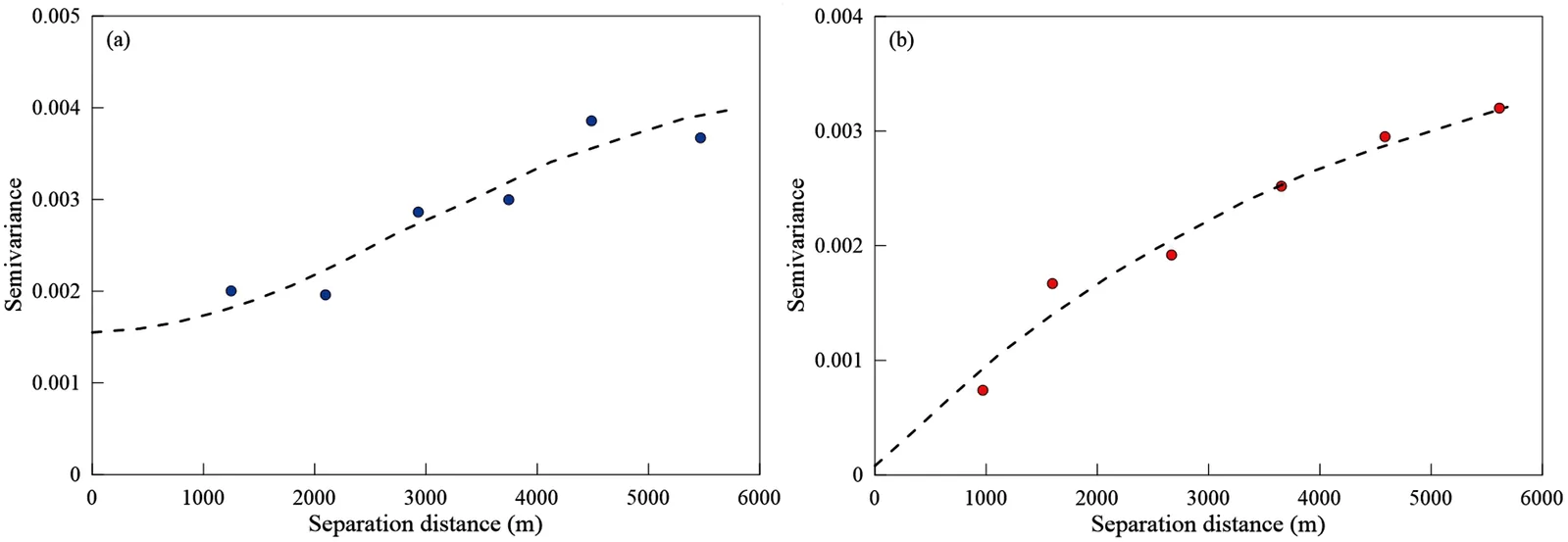
The semi-variogram models including (a) Gaussian and (b) Spherical of fractal dimension.

Subsequently, semi-variogram models were fitted using GS + 10.0 software to quantify the spatial structure of fractal dimensions at different depths. Four common models, such as linear, spherical, exponential, and Gaussian were tested, and the optimal model for each depth was selected based on the best fit (Table 3). The fitted models included both spherical and Gaussian types, as illustrated in Fig 3.

**Table 3:** Semi-variogram models and parameters of soil fractal dimension for different depths.

Depth (cm)	Optimal models	Nugget (C_0_)	Sill (C_0_ + C)	C_0_/(C_0_ + C)	Range (m)	Determination coefficient (R^2^)	Residual sum of squares (RSS)
0-20	Spherical	0.00018	0.0016	0.102	1230.0	0.84	2.67 × 10^−8^
20-40	Gaussian	0.00005	0.0022	0.021	2140.0	0.38	3.79 × 10^−7^
40-60	Gaussian	0.0016	0.0043	0.363	6824.3	0.79	8.49 × 10^−7^
60-80	Spherical	0.00021	0.0042	0.023	1850.0	0.57	3.42 × 10^−6^
80-100	Spherical	0.0022	0.0059	0.373	5300.0	0.72	1.73 × 10^−6^
100-120	Spherical	0.00046	0.0027	0.170	5210.0	0.79	4.61 × 10^−7^
120-140	Gaussian	0.0014	0.0066	0.218	12643.9	0.81	6.89 × 10^−7^
140-160	Gaussian	0.0018	0.0044	0.404	9075.9	0.57	1.41 × 10^−6^
160-180	Gaussian	0.0016	0.0118	0.134	17580.3	0.86	4.45 × 10^−7^
180-200	Gaussian	0.0025	0.0052	0.481	2078.5	0.76	5.71 × 10^−7^
200-220	Spherical	0.0020	0.0050	0.396	1040.0	0.10	7.60 × 10^−5^
220-240	Gaussian	0.0020	0.0040	0.398	1040.0	0.37	9.02 × 10^−5^

The nugget to sill ratio C_0_/(C_0_ + C) was used to assess the degree of spatial dependence. The values ranged from 2.3% to 48.1%, with most layers exhibiting ratios below 25%, indicating strong spatial dependence. The spatial correlation range varied significantly across depths, from 1,040 m to 17,580 m, suggesting that the spatial structure of soil fractal dimensions is influenced by both local heterogeneity and broader-scale environmental factors.

### 3.4. Spatial distribution of soil fractal dimension

**Fig 4:**
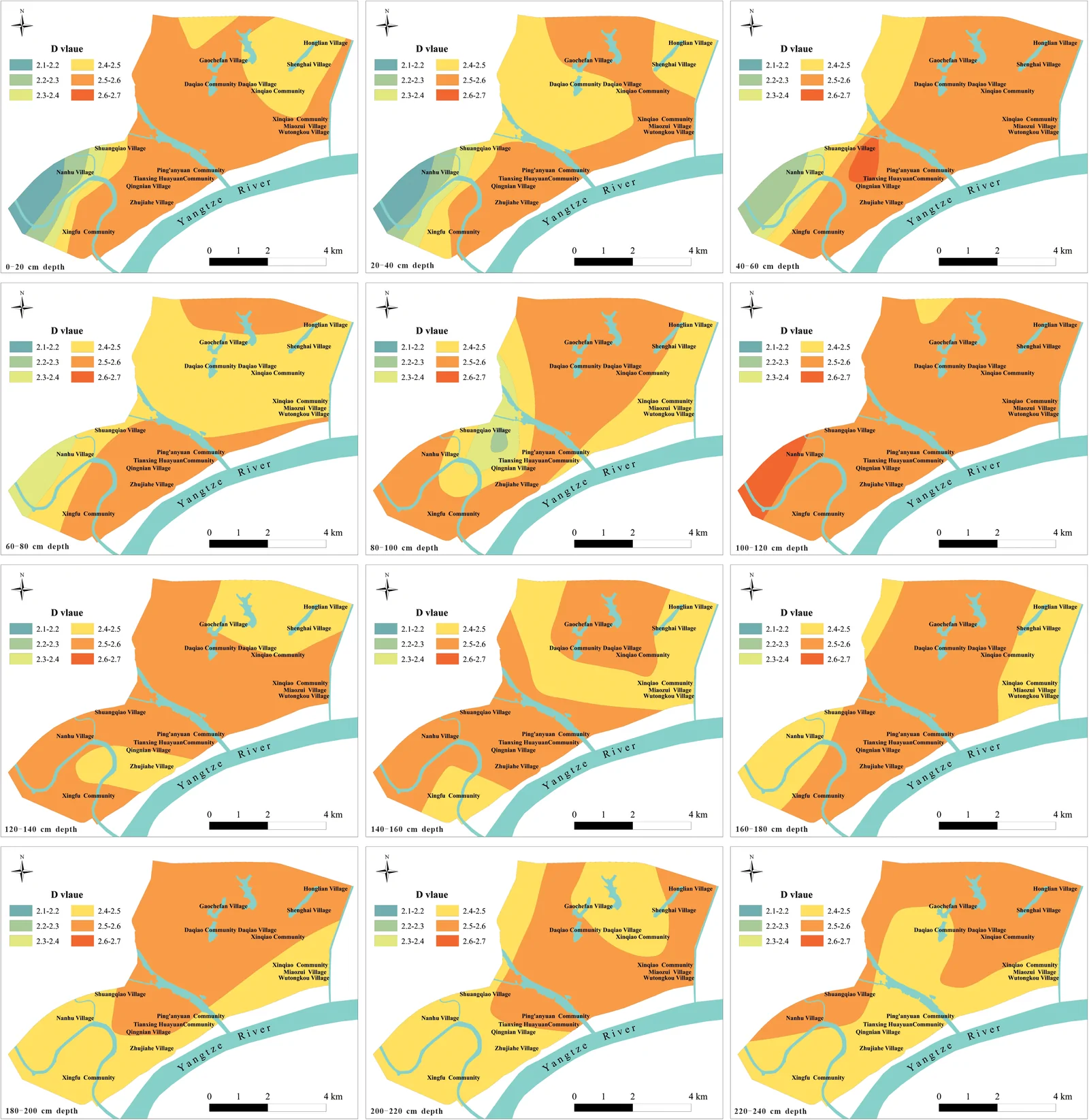
Spatial distribution maps of soil fractal dimensions at different depths.

The spatial distribution of soil fractal dimensions (D values) from 0 to 240 cm depth was derived by interpolation of measured data, as presented in Fig 4. In the upper 70 cm of the soil profile, D values show strong spatial variation (2.07–2.71), which is mainly attributed to Quaternary fill deposits and intensive human activities in the study area.

The spatial pattern of D values is relatively consistent within the 0–70 cm depth range: low values are concentrated in the southwestern region, and high values occur in the northeast. At 70 cm depth, high-D zones are mainly distributed along the Yangtze and Sheshui Rivers, where fluvial sedimentation enriches fine particles (silt and clay) and raises fractal dimensions; in contrast, the southwestern zones have higher sand content and thus lower D values. These results indicate that the spatial heterogeneity of soil fractal dimensions in the study area is jointly controlled by natural sedimentary processes and anthropogenic disturbance, and can directly reflect the spatial differentiation of soil particle composition.

At the 70–80 cm depth, the spatial distribution becomes more uniform, with D values showing minimal variation and soils dominated by silty clay. Between 80 and 100 cm, a distinct pattern emerges: higher D values are observed along the periphery of the study area, while lower values appear in the central zone. Below 100 cm, the D values stabilize within a narrower range (2.42–2.63), with generally higher values near the Yangtze River, reflecting the influence of long-term fluvial deposition and lithological consistency in deeper layers.

### 3.5. Three-dimensional spatial visualization of soil fractal dimensions

**Fig 5:**
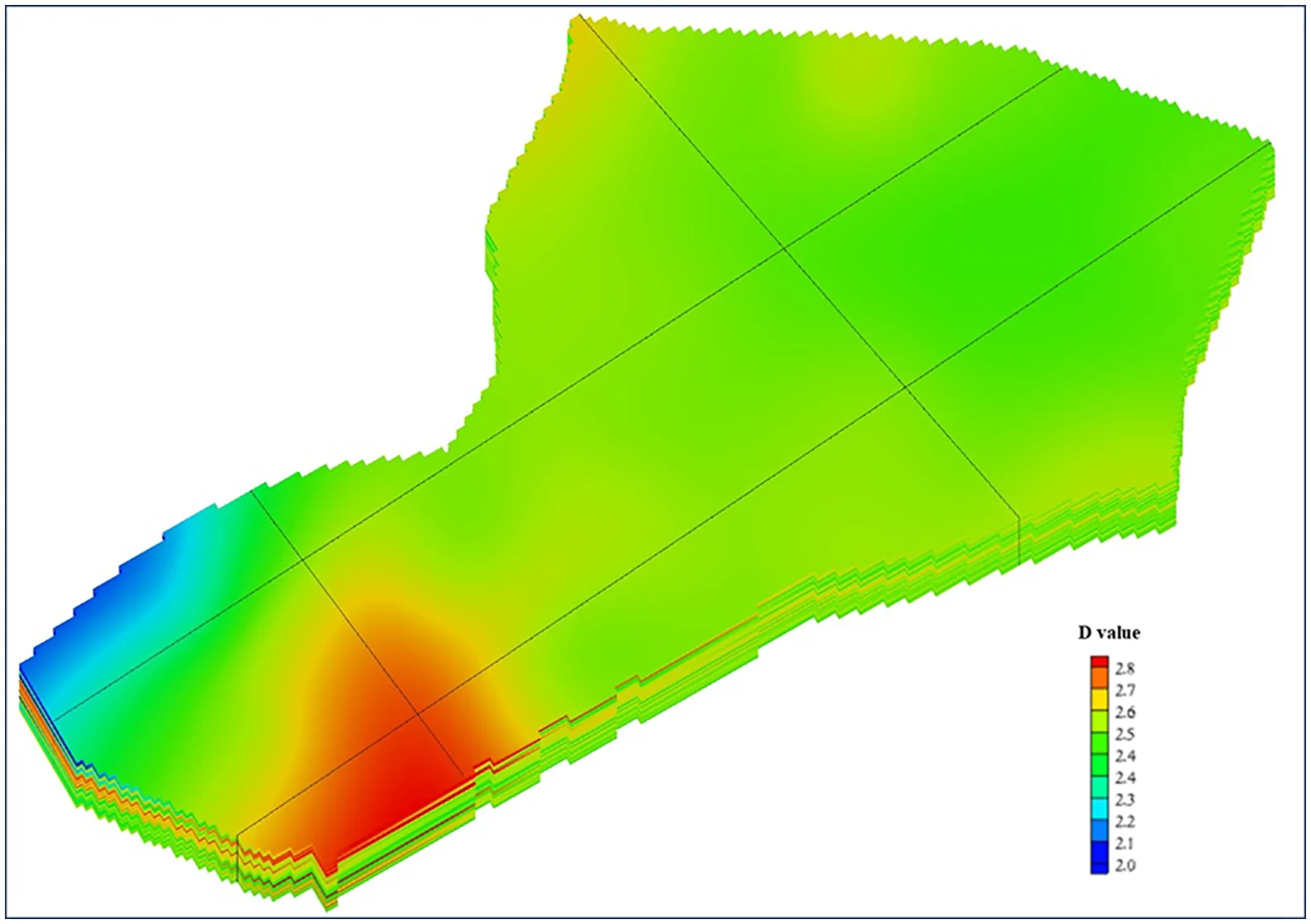
Three-dimensional spatial distribution of D values. The black lines represent the cross-section line.

**Fig 6:**
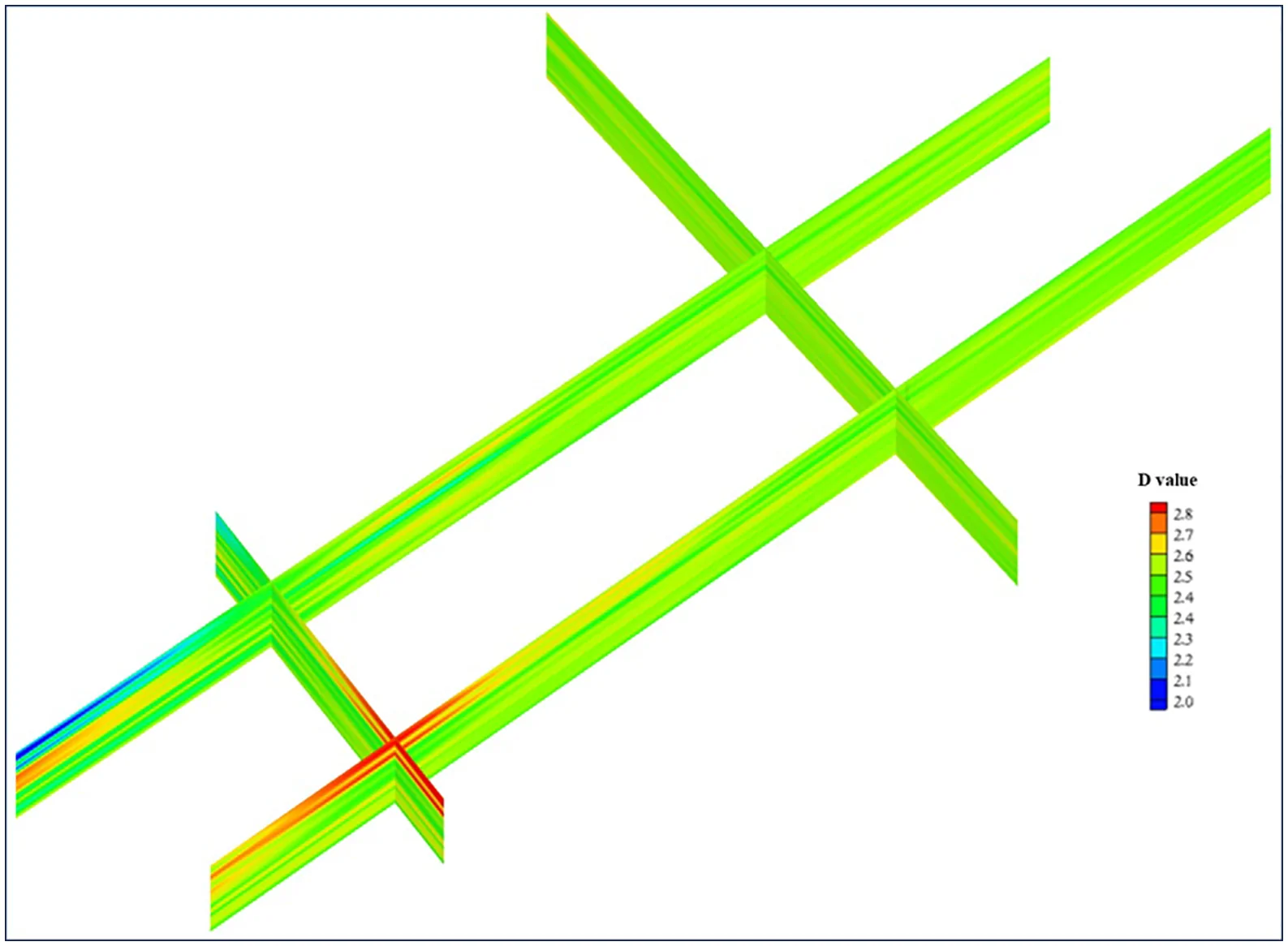
Fence diagram of soil D values. The cross-section lines are shown in Fig 5.

Soil particle compositions were analyzed using high-resolution sampling at 10 cm intervals from 0 to 240 cm depth, and the fractal dimension was calculated for each layer. The spatial variability of soil fractal dimensions at different depths was assessed using semi-variogram models, and their spatial distribution patterns were visualized through spatial interpolation methods. Based on the calculated D values of each layer, a three-dimensional structural model of soil fractal dimensions was constructed using GMS (Groundwater Modeling System) 10.0 software, as presented in Fig 5. Combined with the fence diagram in Fig 6, the three-dimensional model clearly exhibits the spatial distribution of soil fractal dimensions throughout the study area.

As shown in Figs 5 and 6, the surface water systems, including Sheshui, Zhujia, Fu, and Yangze Rives, are well-developed in the southwestern part (such as Xingfu, Zhujiahe, and Ping’anyuan Communities) of the study area, resulting in a relatively wide range of soil D values. In contrast, the northeastern region exhibits a relatively narrower range in D values. Soil fractal dimensions become more uniform with increasing depth across the region. This pattern may be caused by the diminishing influence of regional lithology variation and artificial fill with depth. The three-dimensional model of D values effectively reflects the spatial distribution of soil texture, and provides a robust foundation for the planning and design of sponge cities.

### 3.6. Fractal modeling and prediction of soil water characteristic curves (SWCCs)

The fractal dimension of soil PSD serves as a key indicator of soil texture and structure, and is closely linked to hydraulic properties such as water retention and permeability. Traditional methods for measuring soil water characteristic curves (SWCCs) are labor-intensive and time-consuming. To address this, fractal-based models, such as the Tyler-Wheatcraft, Brooks-Corey, and van Genuchten models have been developed to estimate SWCCs from soil fractal dimensions [9,12,32,33]. Based on soil particle mass distribution data, Bird et al. (2000) [34] proposed a widely adopted fractal model for understanding the SWCCs by integrating the pore-solid fractal (PSF) framework with the Young–Laplace equation. As a generalized fractal construct introduced by Perrier et al. (1999) [35], the PSF model delineates soil complexity by dividing into three proportional components: pore set (P), solid set (S), and fractal set (F), with their proportions governed by *P* + *S* + *F* = 1. Assuming a fractal entity with characteristic length L, composed of *N*_*F*_ sub-regions (*γ*_*F*(1)_) and analogously structured pore (*Np*, *γ*_*P*(1)_) and soil (*N*_*S*_, *γ*_*S*(1)_) configurations, Bird et al. (2000) [34] advanced the fractal model to predict the SWCCs as follows:


$$\begin{array}{c} \theta =\Phi -\frac{P}{P+S}\left[1-{\left(\frac{\psi }{\psi_{a}}\right)}^{D-3}\right] \end{array}$$ (4)

$$\begin{array}{c} \Phi =\frac{P}{P+S} \end{array}$$ (5)

where *θ* represent the volumetric water content, *θ*_*s*_ represent the saturated volumetric water content, Φ is soil total porosity, D is fractal dimension, ψ is the matrix suction, $\psi_{a}$ is the air-entry value of soil.

**Fig 7:**
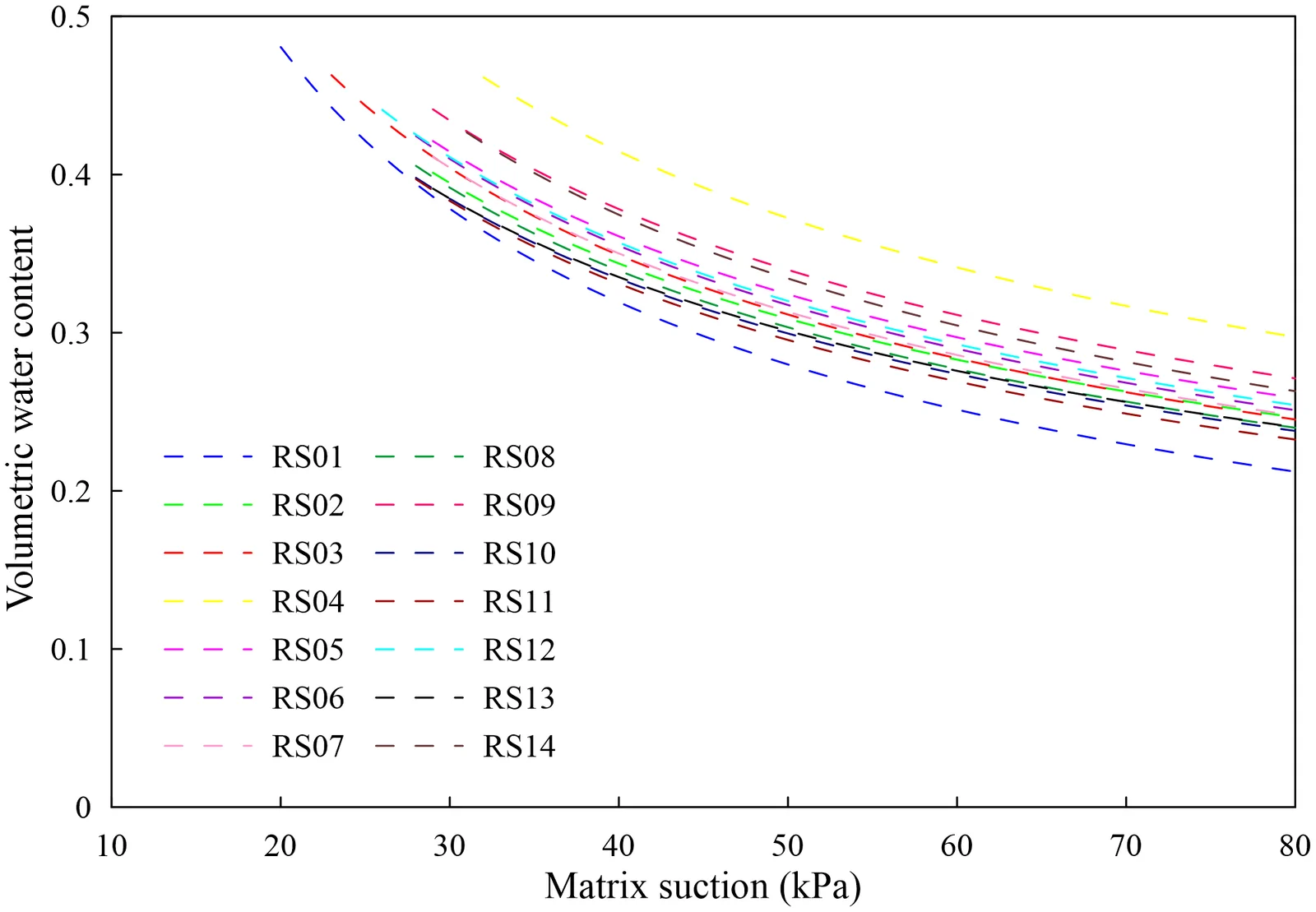
Soil water characteristics curves of 14 soil samples predicted by using fractal model.


According to the PSF model, a generalized fractal SWCCs model [34,36] was employed to predict SWCCs for representative soil samples (Fig 7). This model is expressed as follows:

$$\begin{array}{c} \frac{\theta }{\theta_{s}}={\left(\frac{\psi }{\psi_{a}}\right)}^{D-3} \end{array}$$ (6)

where *θ* represent the volumetric water content, *θ*_*s*_ represent the saturated volumetric water content, ψ is the matrix suction, $\psi_{a}$ is the air-entry value of soil.

For numerical prediction of soil water retention curves using the above fractal model, matrix suction was set as a gradient series covering 0–1500 kPa, consistent with the measurement range of standard pressure plate tests, to generate the complete water retention curve.

The model incorporates three key parameters: saturated volumetric water content (*θ*_*s*_), air-entry value (*ψ*_*a*_), and fractal dimension (D). The saturated water content was measured in the field using MPKit406. Air-entry values were assigned based on soil texture classification, with empirical values adopted from a classic soil hydraulic parameter reference dataset [37]. Values for the four texture classes present in the study area are listed in Table 4. The predicted SWCCs show that volumetric water content decreases with increasing matrix suction. Rapid changes occur at low suction levels (<30 kPa), primarily due to capillary forces, while more gradual changes are observed at higher suction levels, where adsorption dominates [11,35].

**Table 4:** Empirical air-entry values for different soil texture.

Soil texture	Clay	Silty clay	Silt	Sand
Value (kPa)	37.30	29.17	20.00	7.26

The predicted SWCCs show distinct differences in water retention capacity among soil samples. For example, sample RS01, with a silt texture and the lowest fractal dimension (D = 2.32) among the tested samples, exhibits the weakest water retention capacity, with volumetric water content decreasing rapidly as matric suction increases. In contrast, sample RS04, with a silty clay texture and the highest fractal dimension (D = 2.61), maintains the highest volumetric water content across the full suction range, showing the strongest water retention capacity, which verified that fractal dimension characterizes pore size distribution heterogeneity and can reliably reflect soil water retention behavior [11,38].

## 4. Discussion

### 4.1. Engineering implications of soil texture and fractal characteristics for sponge city construction

The combined analysis of soil particle size distribution (PSD), fractal dimension (D), and their spatial variability provide a quantitative basis for characterizing subsurface soil heterogeneity in the study area, which is essential for sponge city infrastructure planning and design. The study area is dominated by silt with moderate clay and low sand content, representing typical fine-textured soils. Consistent with established conclusions from soil physics and urban hydrology studies, fine-textured soils generally feature high water retention capacity but limited infiltration rate, as fine particles form small pore throats that retain water but restrict rapid seepage [31,35]. This textural property favors soil water storage and rainwater retention under light and moderate rainfall. However, under the intense rainfall events characteristic of Wuhan’s subtropical monsoon climate, the limited infiltration capacity may fail to match high rainfall intensity, potentially increasing surface runoff generation without targeted sponge facility design. This aligns with findings from previous sponge city soil hydrology studies in central China. It should be noted that actual soil hydraulic performance is also modulated by soil structure, bulk density and anthropogenic compaction, not solely by particle size composition.

The high spatial variability in sand content (CV = 71.21%) indicates significant localized differences in infiltration potential, while the low variability in silt (CV = 8.73%) and moderate variability in clay (CV = 20.27%) reflect a relatively stable fine-grained matrix in the upper soil layers. The spatial distribution map of soil fractal dimensions derived in this study can be directly used for hydrological functional zoning in sponge city planning, which supports targeted facility layout and reduces redundant detailed geological survey work. Zones with higher sand content and lower D values are suitable for infiltration-based facilities such as permeable pavements and infiltration trenches, whereas silt- and clay-dominated zones with higher D values should adopt storage-oriented schemes including bioretention cells and supporting drainage pipelines. Fractal dimension provides a synthetic quantitative indicator for soil texture complexity, compensating for the limitation that traditional single grain-fraction parameters cannot fully characterize soil structural and hydraulic properties [9,22]. The distinct vertical pattern of D values—high heterogeneity in the upper 100 cm and strong homogeneity in deeper layers—stems from differences in soil formation processes: shallow soils have been extensively altered by Quaternary artificial fill and intensive anthropogenic activities, resulting in discontinuous textural structures; whereas soils below 100 cm consist mainly of undisturbed natural fluvial-lacustrine deposits with stable sedimentary sequences.

This vertical differentiation has critical implications for sponge city hydrological modeling. Most existing urban hydrological models adopt simplified vertically homogeneous soil parameters, which introduces notable deviations in infiltration and storage calculations. The vertical D-value pattern identified in this study can support layered parameterization in hydrological models, improving the accuracy of rainwater runoff simulation while reducing unnecessary deep soil hydraulic testing workload.

Geostatistical modeling yields quantitative spatial dependence parameters for soil fractal dimensions at each depth. Most layers exhibit strong to moderate spatial dependence (C_0_/(C_0_ + C) < 25%), with spatial correlation ranges varying from 1,040 m to 17,580 m. These parameters provide actionable quantitative criteria for optimizing field sampling schemes and sponge facility layout. For shallow heterogeneous layers (0–100 cm) with short correlation ranges, sampling intervals should be limited to 2–3 km in detailed site surveys to capture local textural heterogeneity. For deeper layers (100–240 cm) with longer correlation ranges, sampling density can be appropriately reduced to lower geological survey costs while maintaining spatial representativeness. For engineering design, the spatial autocorrelation scale can guide the deployment spacing of infiltration and storage facilities, avoiding design deviation caused by assuming uniform soil properties across large areas, and improving the accuracy of regional rainwater runoff simulation.

### 4.2. Fractal SWCC modeling and its sponge city design implications

The successful application of fractal models to predict SWCCs demonstrates their potential as a practical tool for estimating soil hydraulic behavior, particularly in deep vadose zones where direct measurements are often impractical. By linking fractal dimension to key hydraulic parameters, the model provides a rapid and scalable approach to characterize water retention and infiltration capacity across diverse soil textures.

To evaluate the reliability of the fractal model, a subset of soil samples was selected for direct SWCC measurements using laboratory pressure plate apparatus. The comparison between measured and predicted SWCCs showed strong agreement, with a coefficient of determination (R^2^) exceeding 0.90 in most cases [10,38,39]. This high level of accuracy confirms the model’s robustness and its applicability for estimating soil hydraulic properties in data-scarce environments.

The observed positive relationship between fractal dimension and water retention capacity is consistent with conclusions from soil fractal hydrology studies in different climatic and geological regions worldwide [18,32,35]. This convergence is reflected in the consistent trend that higher fine-particle content raises fractal dimension and correspondingly enhances soil water-holding capacity by increasing the proportion of micropores. Most existing studies are limited to topsoil within 50 cm depth in agricultural or natural areas, while this study extends fractal analysis to 240 cm deep urban vadose zones influenced by artificial fill and fluvial deposition. The stable performance of the fractal SWCCs model in deep layered soils verifies the generalizability of fractal theory in complex urban sedimentary environments, and fills the gap of efficient hydraulic parameter estimation for deep urban soils.

### 4.3. Optimization of sponge city facility design based on fractal characteristics

Wuhan City, located in the middle-lower reaches of the Yangtze River, is characterized by complex Quaternary depositional sequences that exert primary control on the spatial distribution of soil hydraulic properties. The regional lithology mainly includes silty clay, silt and clay with distinct depth-dependent variations. The fractal dimension (D) of soil particles, which quantifies the complexity of pore structure, shows significant depth-stratified patterns (Table 2): from 0–20 cm (mean D = 2.49) to 220–240 cm (mean D = 2.51), with fluctuations reflecting changes in depositional environments. The soil water characteristic curve (SWCC), parameterized via fractal-based empirical relations (e.g., van Genuchten model), links matric suction (ψ) to volumetric water content (θ).

By integrating soil particle fractal dimensions with SWCC modeling, distinct lithological units can be classified based on their water retention and infiltration behavior, providing a scientific basis for zoned Sponge City engineering designs. The ultimate goal of sponge city construction is to enhance urban resilience by improving rainwater infiltration, storage, and purification [7], and the fractal dimension of soil PSD offers a scientifically grounded parameter for guiding the selection and spatial deployment of sponge infrastructure. In general, high D values, indicative of fine-textured, compact soils, suggest strong water retention but limited infiltration, making such areas suitable for retention-based facilities like rain gardens, grass swales, or constructed wetlands. Conversely, low D values, associated with coarser textures and higher permeability, are more appropriate for infiltration-based systems such as permeable pavements.

In terms of detailed design optimization, high-D zones with fine-grained soils have low hydraulic conductivity (inferred from low α in SWCC), so underdrains or overflow weirs are required to prevent waterlogging and ensure vegetation health, and the depth of the depression should be increased (e.g., 30–50 cm) to compensate for slower infiltration. For low-D zones with well-drained soils where rapid infiltration is desired, soil amendment (e.g., adding clay or organic matter to increase D and water retention) can be adopted to avoid excessive runoff during heavy rainfall; alternatively, pre-treatment filters (e.g., gravel trenches) can be installed to retain sediments before infiltration.

Specifically, three typical depositional units in the study area correspond to more targeted engineering design schemes. First, modern floodplain silt of the Yangtze River typically exhibits a relatively low fractal dimension (D ≈ 2.3–2.4). The resulting SWCC is characterized by the high *α* parameter (low air-entry value) and the low *n* parameter (narrow pore-size distribution), indicating rapid initial infiltration but limited water retention capacity. Consequently, these deposits are optimally utilized as base courses for permeable pavements or in rapid infiltration trenches. However, engineering designs must account for the risk of perched water tables forming atop underlying impermeable strata; thus, underdrains or interception drains are recommended to divert excess gravitational water. Second, old clay terraces represent highly weathered Pleistocene deposits with a higher fractal dimension (D ≈ 2.6–2.7). The corresponding SWCC features a low *α* (high air-entry value) and a high *n* (broad pore-size distribution), signifying strong matric potential and excellent water storage capacity, albeit with slow gravity drainage. These soils are ideal for bio-retention cells and sunken green spaces where long-term storage is prioritized. To prevent prolonged waterlogging and hypoxic root conditions, engineering interventions, such as perforated underdrains and controlled overflow weirs calibrated to a critical saturation degree (Sr,crit≈0.85) are strictly necessary. Third, lacustrine deposits, prevalent around Wuhan’s numerous urban lakes, present the most heterogeneous conditions with high and variable D values. Their SWCCs are notably wide with pronounced hysteresis, reflecting the interbedded nature of silty clay and fine sand layers deposited in quiet water environments. This vertical anisotropy facilitates capillary migration and reduces the effective recharge capacity during consecutive storm events. Therefore, a layered structural design is imperative: coarse-grained layers (D < 2.3) should be placed in upper zones for conduction, while fine-grained layers (D > 2.6) serve as lower storage zones, separated by a capillary break (e.g., a 15 cm crushed stone layer). High-resolution numerical simulations (e.g., HYDRUS-1D) incorporating multi-layered SWCCs are recommended to accurately predict the stormwater mitigation efficiency of LID facilities in these areas.

By integrating the three-dimensional D value model with the fractal SWCC predictions, this study provides a spatially explicit framework for facility selection at the site scale. For example, in Xingfu and Zhujiahe Communities (samples RS02, RS03, RS05), low D values and high sand content suggest suitability for permeable pavements. In contrast, areas near the Fu River (e.g., RS04) with high D values and clay-rich soils are better suited for retention-focused designs. In transitional zones such as Nanhu Village (RS01) and the southern region (RS06–RS12), mixed strategies combining infiltration and retention measures may be optimal.

Compared with traditional laboratory pressure plate tests, this fractal-based approach can directly derive continuous hydraulic parameters for all depth layers from conventional PSD test data, significantly reducing the cost and period of deep soil hydraulic testing. For large-scale urban planning with limited survey data, this method provides an efficient technical path to obtain spatially distributed soil hydraulic parameters, and is applicable to similar alluvial plain cities with Quaternary fine-grained deposits globally.

These findings demonstrate that fractal analysis of soil PSD, combined with SWCC modeling, can significantly enhance the precision and efficiency of sponge city planning. By providing a rapid, cost-effective method for estimating key hydraulic parameters, this approach supports data-driven decision-making in urban water management and ecological infrastructure design.

## 5. Conclusion

This study applies fractal theory and geostatistics to analyze the spatial variability of soil particle size distribution (PSD) and its hydrological relevance in Wuhan’s sponge city area. A three-dimensional model visualizes the distribution of soil fractal dimensions, while a fractal-based method estimates key hydraulic parameters, including soil water characteristic curves (SWCCs). These results support efficient evaluation of soil infiltration and retention capacity, providing a scientific basis for site-specific low-impact development (LID) planning. The main findings are as follows:

1. High-resolution sampling and laser diffraction analysis revealed that soil fractal dimensions ranged from 2.07 to 2.71, with moderate to strong spatial dependence influenced by climate, topography, and lithology.
2. A predictive model based on soil fractal dimensions accurately estimated SWCCs, with validation results showing over 90% agreement with measured data. Higher D values indicated stronger water retention and lower permeability, while lower D values corresponded to higher infiltration potential.
3. By combining spatial modeling and hydraulic predictions, the study proposed differentiated LID strategies. Permeable pavements are recommended for sandy, low-D areas, while rain gardens and bioswales are more suitable for clay-rich, high-D zones. This approach supports data-driven, site-specific sponge city planning.

## Supporting information

S1 Table Analysis results of soil samples taken from the study area. (PDF)
