# Supplementary material for: Spatial variability of soil texture fractal dimension and its application in sponge city construction
Source: PLoS One. 2026 Sep 21;21(9):e0357100. doi: 10.1371/journal.pone.0357100 (PMC13592644; doi:10.1371/journal.pone.0357100)
Supplement: Supplementary file 1 [file pone.0357100.s001.pdf]

Table S1. Analysis results of soil samples taken from the study area.

Samples ID	Longitude	Latitude	Depth (cm)	Particle composition (%)				
				0.50-0.25 (mm)	0.25-0.075 (mm)	0.075-0.005 (mm)	0.005-0.002 (mm)	< 0.002 (mm)
RS01	532067.23	3396218.58	0-10		1.6	75.5	16.9	6.0
RS01	532067.23	3396218.58	10-20		0.3	89.7	6.9	3.1
RS01	532067.23	3396218.58	20-30		11	77.9	8.1	3
RS01	532067.23	3396218.58	30-40		4.8	81.5	7.5	6.2
RS01	532067.23	3396218.58	40-50		4	74.8	10.6	10.6
RS01	532067.23	3396218.58	50-60		2.8	84.9	9.2	3.1
RS01	532067.23	3396218.58	60-70		3.0	76.7	15.2	5.1
RS01	532067.23	3396218.58	70-80		11.5	62.6	10	15.9
RS01	532067.23	3396218.58	80-90		12.4	56.1	9.8	21.7
RS01	532067.23	3396218.58	90-100		14.7	62.3	7.4	15.6
RS01	532067.23	3396218.58	100-110	1.4	12.4	57.9	11.3	17
RS01	532067.23	3396218.58	110-120		11.3	58.2	10.5	20
RS01	532067.23	3396218.58	120-130		16.3	58	10.1	15.6
RS01	532067.23	3396218.58	130-140		5.6	54	17.2	23.2
RS01	532067.23	3396218.58	140-150		12.1	57.3	10.2	20.4
RS02	532717.24	3395183.39	0-10					
RS02	532717.24	3395183.39	10-20		8.6	64.9	8.5	18
RS02	532717.24	3395183.39	20-30		7.9	70.9	8.4	12.8
RS02	532717.24	3395183.39	30-40		4	71.6	12.9	11.5
RS02	532717.24	3395183.39	40-50		8.8	66.9	5.8	18.5
RS02	532717.24	3395183.39	50-60		8.1	55.3	17.4	19.2
RS02	532717.24	3395183.39	60-70		9.2	58.7	17.6	14.5
RS02	532717.24	3395183.39	70-80		7.7	65.8	12.6	13.9
RS02	532717.24	3395183.39	80-90		12.7	63.5	7.4	16.4
RS02	532717.24	3395183.39	90-100		10.7	65.7	6.5	17.1
RS02	532717.24	3395183.39	100-110		8.6	54.8	18.8	17.8
RS02	532717.24	3395183.39	110-120		8.9	62.9	9.0	19.2
RS02	532717.24	3395183.39	120-130		12.9	55.9	13.6	17.6
RS02	532717.24	3395183.39	130-140		3.1	66.9	13.8	16.2
RS02	532717.24	3395183.39	140-150		9.6	64.2	7.9	18.3
RS02	532717.24	3395183.39	150-160		8.1	61.7	15.6	14.6
RS02	532717.24	3395183.39	160-170		11.4	68.6	8.2	11.8
RS02	532717.24	3395183.39	170-180		11.3	59.8	14.2	14.7
RS02	532717.24	3395183.39	180-190		4.2	73.7	5.4	16.7
RS02	532717.24	3395183.39	190-200		6.2	65.1	16.1	12.6
RS02	532717.24	3395183.39	200-210		12.4	64	7.5	16.1
RS02	532717.24	3395183.39	210-220		3.0	78	6.7	12.3
RS02	532717.24	3395183.39	220-230		8.0	66.6	10.9	14.5
RS02	532717.24	3395183.39	230-240		12.8	66.6	6.8	13.8

Table S1 (continued)

Samples ID	Longitude	Latitude	Depth (cm)	Particle composition (%)				
				0.50-0.25 (mm)	0.25-0.075 (mm)	0.075-0.005 (mm)	0.005-0.002 (mm)	< 0.002 (mm)
RS03	533439.46	3396001.91	0-10		8.4	53.2	19.2	19.2
RS03	533439.46	3396001.91	10-20		4.5	63.9	16.3	15.3
RS03	533439.46	3396001.91	20-30		9.8	66.3	6.7	17.2
RS03	533439.46	3396001.91	30-40		12.0	64.0	11.8	12.2
RS03	533439.46	3396001.91	40-50		9.7	67.2	11.1	12.0
RS03	533439.46	3396001.91	50-60		4.7	65.2	13.1	17.0
RS03	533439.46	3396001.91	60-70		9.1	56.9	17.8	16.2
RS03	533439.46	3396001.91	70-80		7.8	59.5	17.6	15.1
RS03	533439.46	3396001.91	80-90		9.5	61.5	15.2	13.8
RS03	533439.46	3396001.91	90-100		3.9	75.6	9.4	11.1
RS03	533439.46	3396001.91	100-110		11.9	65.7	6.1	16.3
RS03	533439.46	3396001.91	110-120		4.5	68.4	11.5	15.6
RS03	533439.46	3396001.91	120-130		7.0	75.9	5.0	12.1
RS03	533439.46	3396001.91	130-140		8.0	63.2	16.1	12.7
RS03	533439.46	3396001.91	140-150		5.3	69.6	8.6	16.5
RS03	533439.46	3396001.91	150-160		12.7	65.5	8.8	13.0
RS03	533439.46	3396001.91	160-170		6.8	72.5	9.0	11.7
RS03	533439.46	3396001.91	170-180		12.7	52.6	19.5	15.2
RS03	533439.46	3396001.91	180-190		8.9	70.4	5.2	15.5
RS03	533439.46	3396001.91	190-200		3.9	75.8	5.8	14.5
RS03	533439.46	3396001.91	200-210		4.7	73.0	12.0	10.3
RS03	533439.46	3396001.91	210-220		7.4	69.4	12.0	11.2
RS04	534679.28	3397542.65	0-10		3	55.3	24.7	17
RS04	534679.28	3397542.65	10-20		13	44.6	19.9	22.5
RS04	534679.28	3397542.65	20-30		37.7	36.1	10.9	15.3
RS04	534679.28	3397542.65	30-40		11.5	52.0	22.5	14.0
RS04	534679.28	3397542.65	40-50		17.2	42.9	24.2	15.7
RS04	534679.28	3397542.65	50-60		11.2	60.2	9.8	18.8
RS04	534679.28	3397542.65	60-70		7.1	63.2	10.6	19.1
RS04	534679.28	3397542.65	70-80		10.7	59.6	10.2	19.5
RS04	534679.28	3397542.65	80-90		25.2	56.7	5.7	12.4
RS04	534679.28	3397542.65	90-100		8.1	59.3	11.4	21.2
RS04	534679.28	3397542.65	100-110		3.1	58.0	14.0	24.9
RS04	534679.28	3397542.65	110-120		6.8	58.2	15.1	19.9
RS04	534679.28	3397542.65	120-130		8.7	62.1	7.5	21.7
RS04	534679.28	3397542.65	130-140		13.6	56.5	10.6	19.3
RS04	534679.28	3397542.65	140-150		9.3	63.1	9.5	18.1
RS04	534679.28	3397542.65	150-160		12.6	60	11.9	15.5
RS04	534679.28	3397542.65	160-170		12.3	58.6	8.0	21.1

Table S1 (continued)

Samples ID	Longitude	Latitude	Depth (cm)	Particle composition (%)				
				0.50-0.25 (mm)	0.25-0.075 (mm)	0.075-0.005 (mm)	0.005-0.002 (mm)	< 0.002 (mm)
RS04	534679.28	3397542.65	170-180		7.2	58.2	11.8	22.8
RS04	534679.28	3397542.65	180-190		11.1	59.8	8.9	20.2
RS04	534679.28	3397542.65	190-200		10.1	58.9	12.9	18.1
RS04	534679.28	3397542.65	200-210		9.9	58.0	12.2	19.9
RS04	534679.28	3397542.65	210-220		10.5	57.4	9.9	22.2
RS04	534679.28	3397542.65	220-230		12.5	57.2	10.6	19.7
RS04	534679.28	3397542.65	230-240		7.9	56.6	15.8	19.7
RS05	535798.72	3396326.91	0-10		7.9	63.3	14.6	14.2
RS05	535798.72	3396326.91	10-20		11.5	62.9	9.3	16.3
RS05	535798.72	3396326.91	20-30		7.3	56.3	9.5	26.9
RS05	535798.72	3396326.91	30-40		3.5	74	7.5	15
RS05	535798.72	3396326.91	40-50		12.4	65.7	7.2	14.7
RS05	535798.72	3396326.91	50-60		6.1	63.8	14	16.1
RS05	535798.72	3396326.91	60-70		1.1	67.1	9.1	22.7
RS05	535798.72	3396326.91	70-80		9.3	58.2	15.7	16.8
RS05	535798.72	3396326.91	80-90		13.5	53.9	19.3	13.3
RS05	535798.72	3396326.91	90-100		9.0	62.2	15.0	13.8
RS05	535798.72	3396326.91	100-110		10.5	52.9	18.3	18.3
RS05	535798.72	3396326.91	110-120		8.1	61.8	13.2	16.9
RS05	535798.72	3396326.91	120-130		8.9	56.1	18.8	16.2
RS05	535798.72	3396326.91	130-140		7.0	72.4	8.1	12.5
RS05	535798.72	3396326.91	140-150		6.3	54.0	20.2	19.5
RS05	535798.72	3396326.91	150-160		7.9	71.3	6.9	13.9
RS05	535798.72	3396326.91	160-170		3.9	56	22.2	17.9
RS05	535798.72	3396326.91	170-180		7.8	64	13.1	15.1
RS05	535798.72	3396326.91	180-190		6.6	72.3	6.6	14.5
RS05	535798.72	3396326.91	190-200		7.5	66.0	10.4	16.1
RS05	535798.72	3396326.91	200-210		11.6	64.4	10.4	13.6
RS05	535798.72	3396326.91	210-220		8.0	67.7	10.2	14.1
RS05	535798.72	3396326.91	220-230		8.9	66.4	9.2	15.5
RS05	535798.72	3396326.91	230-240		9.9	67.8	10.9	11.4
RS06	536147.80	3399348.21	0-10		11	58.6	16.7	13.7
RS06	536147.80	3399348.21	10-20		5.5	66.8	6.8	20.9
RS06	536147.80	3399348.21	20-30		11.2	64.8	12.0	12.0
RS06	536147.80	3399348.21	30-40		9.3	71.9	7.2	11.6
RS06	536147.80	3399348.21	40-50		8.8	64.5	10.1	16.6
RS06	536147.80	3399348.21	50-60		11.3	69.2	6.1	13.4
RS06	536147.80	3399348.21	60-70		11.1	72.4	6.4	10.1
RS06	536147.80	3399348.21	70-80		9.4	72.3	4.6	13.7

Table S1 (continued)

Samples ID	Longitude	Latitude	Depth (cm)	Particle composition (%)				
				0.50-0.25 (mm)	0.25-0.075 (mm)	0.075-0.005 (mm)	0.005-0.002 (mm)	< 0.002 (mm)
RS06	536147.80	3399348.21	80-90		15.1	46.9	20.1	17.9
RS06	536147.80	3399348.21	90-100		2.8	77.9	6.9	12.4
RS06	536147.80	3399348.21	100-110		5.3	64.6	11.9	18.2
RS06	536147.80	3399348.21	110-120		10.9	63.1	7.8	18.2
RS06	536147.80	3399348.21	120-130		10.7	56.8	16	16.5
RS06	536147.80	3399348.21	130-140		4.1	64.8	13.8	17.3
RS06	536147.80	3399348.21	140-150		10.8	67.4	6.3	15.5
RS06	536147.80	3399348.21	150-160		7.2	65.6	14.7	12.5
RS06	536147.80	3399348.21	160-170		11.9	54.9	18.3	14.9
RS06	536147.80	3399348.21	170-180		4.4	71.4	9.6	14.6
RS06	536147.80	3399348.21	180-190		8.1	70.7	5.6	15.6
RS06	536147.80	3399348.21	190-200		5.8	63.0	14.6	16.6
RS06	536147.80	3399348.21	200-210		7.4	72.6	4.5	15.5
RS06	536147.80	3399348.21	210-220		3.6	72.5	4.5	19.4
RS06	536147.80	3399348.21	220-230		4.2	68.8	15.1	11.9
RS06	536147.80	3399348.21	230-240		11.1	64.9	13.0	11.0
RS07	538458.92	3398770.43	0-10		6.2	60.3	20.4	13.1
RS07	538458.92	3398770.43	10-20		11	63.5	13	12.5
RS07	538458.92	3398770.43	20-30		10	69.9	7.4	12.7
RS07	538458.92	3398770.43	30-40		11.5	55.8	14.4	18.3
RS07	538458.92	3398770.43	40-50		5.3	70.8	13.1	10.8
RS07	538458.92	3398770.43	50-60		7.4	66.5	12.2	13.9
RS07	538458.92	3398770.43	60-70		5.6	65.4	15.5	13.5
RS07	538458.92	3398770.43	70-80		8.2	70.7	6.2	14.9
RS07	538458.92	3398770.43	80-90		10.5	64.2	9.9	15.4
RS07	538458.92	3398770.43	90-100		7.7	72.4	5.1	14.8
RS07	538458.92	3398770.43	100-110		10.7	60.6	15.0	13.7
RS07	538458.92	3398770.43	110-120		9.7	59.5	17.5	13.3
RS07	538458.92	3398770.43	120-130		4.2	70.6	9.3	15.9
RS07	538458.92	3398770.43	130-140		5.3	54.6	24.8	15.3
RS07	538458.92	3398770.43	140-150		9.6	59	13.1	18.3
RS07	538458.92	3398770.43	150-160		5.8	66.5	14.5	13.2
RS07	538458.92	3398770.43	160-170		4.2	70.0	13.8	12.0
RS07	538458.92	3398770.43	170-180		10.3	63.1	13.6	13.0
RS07	538458.92	3398770.43	180-190		11.0	60.4	14.5	14.1
RS07	538458.92	3398770.43	190-200		10.5	64.2	10.3	15
RS07	538458.92	3398770.43	200-210		9.1	59.4	14.7	16.8
RS07	538458.92	3398770.43	210-220		10.2	68.3	7.0	14.5
RS08	535690.39	3401900.07	0-10		13.5	55.9	10.8	19.8

Table S1 (continued)

Samples ID	Longitude	Latitude	Depth (cm)	Particle composition (%)				
				0.50-0.25 (mm)	0.25-0.075 (mm)	0.075-0.005 (mm)	0.005-0.002 (mm)	< 0.002 (mm)
RS08	535690.39	3401900.07	10-20		5.4	70.4	8.7	15.5
RS08	535690.39	3401900.07	20-30		4.9	77.0	5.6	12.5
RS08	535690.39	3401900.07	30-40		5.3	61.6	20.6	12.5
RS08	535690.39	3401900.07	40-50		5.2	69.0	12.2	13.6
RS08	535690.39	3401900.07	50-60		4.1	68.4	15.0	12.5
RS08	535690.39	3401900.07	60-70		3.6	73.6	8.1	14.7
RS08	535690.39	3401900.07	70-80		4.0	76.8	6.4	12.8
RS08	535690.39	3401900.07	80-90		3.8	68.1	6.4	21.7
RS08	535690.39	3401900.07	90-100		12.4	59.6	14.2	13.8
RS08	535690.39	3401900.07	100-110		6.9	58.9	13.6	20.6
RS08	535690.39	3401900.07	110-120		12.8	65.5	5.2	16.5
RS08	535690.39	3401900.07	120-130		6.3	68.7	9.4	15.6
RS08	535690.39	3401900.07	130-140		12.3	61.3	14.1	12.3
RS08	535690.39	3401900.07	140-150		8.4	63.1	17.1	11.4
RS08	535690.39	3401900.07	150-160		6.9	70.4	7.7	15.0
RS08	535690.39	3401900.07	160-170		8.5	69.9	9.1	12.5
RS08	535690.39	3401900.07	170-180		7.7	62.5	14.2	15.6
RS08	535690.39	3401900.07	180-190		4.0	71.9	11.6	12.5
RS08	535690.39	3401900.07	190-200		9.6	57	16.2	17.2
RS08	535690.39	3401900.07	200-210		9.0	65.8	10.6	14.6
RS08	535690.39	3401900.07	210-220		3.3	69.9	15.0	11.8
RS08	535690.39	3401900.07	220-230		12.6	63.9	7.2	16.3
RS08	535690.39	3401900.07	230-240		0.2	58.8	22.0	19.0
RS09	537122.80	3401779.70	0-10		7.0	66.9	11.7	14.4
RS09	537122.80	3401779.70	10-20		3.1	73.6	10.3	13.0
RS09	537122.80	3401779.70	20-30		4.2	66.9	14.7	14.2
RS09	537122.80	3401779.70	30-40		10.9	56.8	15.1	17.2
RS09	537122.80	3401779.70	40-50		11.5	53.3	22.4	12.8
RS09	537122.80	3401779.70	50-60		10.7	60.1	16.3	12.9
RS09	537122.80	3401779.70	60-70		12.8	61.4	8.9	16.9
RS09	537122.80	3401779.70	70-80		13.0	57.6	16.3	13.1
RS09	537122.80	3401779.70	80-90		8.9	61.1	16.4	13.6
RS09	537122.80	3401779.70	90-100		11.5	65.3	7.4	15.8
RS09	537122.80	3401779.70	100-110		3.6	73.4	7.2	15.8
RS09	537122.80	3401779.70	110-120		4.8	70.3	11.5	13.4
RS09	537122.80	3401779.70	120-130		10.8	51.9	19.5	17.8
RS09	537122.80	3401779.70	130-140		9.6	51.6	22.7	16.1
RS09	537122.80	3401779.70	140-150		11.0	61.8	13.6	13.6
RS09	537122.80	3401779.70	150-160		7.5	62.5	16.2	13.8

Table S1 (continued)

Samples ID	Longitude	Latitude	Depth (cm)	Particle composition (%)				
				0.50-0.25 (mm)	0.25-0.075 (mm)	0.075-0.005 (mm)	0.005-0.002 (mm)	< 0.002 (mm)
RS09	537122.80	3401779.70	160-170		12.6	52.1	19	16.3
RS09	537122.80	3401779.70	170-180		2.9	65.5	18.6	13.0
RS09	537122.80	3401779.70	180-190		6.4	59.9	17.2	16.5
RS09	537122.80	3401779.70	190-200		5.7	72.1	7.4	14.8
RS09	537122.80	3401779.70	200-210		10.5	59.5	12.7	17.3
RS09	537122.80	3401779.70	210-220		6.6	61.0	19.7	12.7
RS09	537122.80	3401779.70	220-230		1.3	56.1	23.0	19.6
RS09	537122.80	3401779.70	230-240		7.1	63.6	12.2	17.1
RS10	539205.21	3400263.03	0-10		7.8	71.3	8.6	12.3
RS10	539205.21	3400263.03	10-20		12.1	58.6	17.2	12.1
RS10	539205.21	3400263.03	20-30		11.7	63.1	11.1	14.1
RS10	539205.21	3400263.03	30-40		11.8	50.4	23.7	14.1
RS10	539205.21	3400263.03	40-50		3.9	68	12.2	15.9
RS10	539205.21	3400263.03	50-60		6.7	65.4	8.0	19.9
RS10	539205.21	3400263.03	60-70		14.2	62.7	5.5	17.6
RS10	539205.21	3400263.03	70-80		3.8	68.8	16.7	10.7
RS10	539205.21	3400263.03	80-90		11.4	57.6	18.2	12.8
RS10	539205.21	3400263.03	90-100		8.8	60.6	15.3	15.3
RS10	539205.21	3400263.03	100-110		13.6	53.6	18.8	14
RS10	539205.21	3400263.03	110-120		8.9	67.3	10.2	13.6
RS10	539205.21	3400263.03	120-130		8.7	62.4	15.9	13.0
RS10	539205.21	3400263.03	130-140		11.7	68.4	5.6	14.3
RS10	539205.21	3400263.03	140-150		6.5	63.2	16.2	14.1
RS10	539205.21	3400263.03	150-160		5.9	63.2	15.2	15.7
RS10	539205.21	3400263.03	160-170		3.7	69.2	14.2	12.9
RS10	539205.21	3400263.03	170-180		6.7	62.6	13.2	17.5
RS10	539205.21	3400263.03	180-190		9.5	62.8	16.5	11.2
RS10	539205.21	3400263.03	190-200		6.5	54	19.8	19.7
RS10	539205.21	3400263.03	200-210		4.7	65.8	18.0	11.5
RS10	539205.21	3400263.03	210-220		12.9	61.9	11.2	14.0
RS10	539205.21	3400263.03	220-230		7.9	60.1	17.8	14.2
RS10	539205.21	3400263.03	230-240		13.8	54.2	14.7	17.3
RS11	540613.55	3398373.21	0-10		8.4	59.6	15.8	16.2
RS11	540613.55	3398373.21	10-20		8.4	67.1	11.1	13.4
RS11	540613.55	3398373.21	20-30		8.4	61.1	13.6	16.9
RS11	540613.55	3398373.21	30-40		5.7	67.7	6.7	19.9
RS11	540613.55	3398373.21	40-50		13.7	64.1	8.5	13.7
RS11	540613.55	3398373.21	50-60		11.5	56.0	17.7	14.8
RS11	540613.55	3398373.21	60-70		9.6	70.4	7.4	12.6

Table S1 (continued)

Samples ID	Longitude	Latitude	Depth (cm)	Particle composition (%)				
				0.50-0.25 (mm)	0.25-0.075 (mm)	0.075-0.005 (mm)	0.005-0.002 (mm)	< 0.002 (mm)
RS11	540613.55	3398373.21	70-80		8	61	16.3	14.7
RS11	540613.55	3398373.21	80-90		11.1	67.3	9.9	11.7
RS11	540613.55	3398373.21	90-100		6.2	65.2	14.0	14.6
RS11	540613.55	3398373.21	100-110		5.3	65.7	15.6	13.4
RS11	540613.55	3398373.21	110-120		9.5	65.4	9.2	15.9
RS11	540613.55	3398373.21	120-130		4.2	55.6	19.2	21.0
RS11	540613.55	3398373.21	130-140		10.2	62.1	15.2	12.5
RS11	540613.55	3398373.21	140-150		11.0	67.7	6.3	15.0
RS11	540613.55	3398373.21	150-160		8.0	67.0	11.3	13.7
RS11	540613.55	3398373.21	160-170		10	68.6	7.9	13.5
RS11	540613.55	3398373.21	170-180		6.3	69.6	9.2	14.9
RS11	540613.55	3398373.21	180-190		10.0	58.2	19.3	12.5
RS11	540613.55	3398373.21	190-200		4.3	76.0	7.7	12.0
RS11	540613.55	3398373.21	200-210		11.6	65.0	6.4	17.0
RS11	540613.55	3398373.21	210-220		2.9	65.8	17.3	14.0
RS11	540613.55	3398373.21	220-230		2.9	76.3	6.8	14.0
RS11	540613.55	3398373.21	230-240		7.0	61.6	18.4	13.0
RS12	538651.51	3401972.29	0-10		8.9	58.8	15.6	16.7
RS12	538651.51	3401972.29	10-20		11.7	68.9	6.3	13.1
RS12	538651.51	3401972.29	20-30		7.2	54.1	21.1	17.6
RS12	538651.51	3401972.29	30-40		13.7	52.5	19.1	14.7
RS12	538651.51	3401972.29	40-50		6.3	63.5	13.5	16.7
RS12	538651.51	3401972.29	50-60		5.6	76.5	2.7	15.2
RS12	538651.51	3401972.29	60-70		3.2	74.9	7.6	14.3
RS12	538651.51	3401972.29	70-80		4.6	77.2	4.3	13.9
RS12	538651.51	3401972.29	80-90		11.2	69	7.5	12.3
RS12	538651.51	3401972.29	90-100		11.8	63.4	8.6	16.2
RS12	538651.51	3401972.29	100-110		14.8	52.9	17.5	14.8
RS12	538651.51	3401972.29	110-120		4.5	60.8	16.0	18.7
RS12	538651.51	3401972.29	120-130		4.4	76.6	5.6	13.4
RS12	538651.51	3401972.29	130-140		11.7	69.2	5.3	13.8
RS12	538651.51	3401972.29	140-150		9.9	58.4	15.1	16.6
RS12	538651.51	3401972.29	150-160		14.4	54.0	15.0	16.6
RS12	538651.51	3401972.29	160-170		8.8	65.9	9.6	15.7
RS12	538651.51	3401972.29	170-180		5.6	74.6	4.2	15.6
RS12	538651.51	3401972.29	180-190		8.5	59.2	19.4	12.9
RS12	538651.51	3401972.29	190-200		4.6	69.1	11.2	15.1
RS12	538651.51	3401972.29	200-210		8.1	72.6	7.4	11.9
RS12	538651.51	3401972.29	210-220		13.5	63.9	5.9	16.7

Table S1 (continued)

Samples ID	Longitude	Latitude	Depth (cm)	Particle composition (%)				
				0.50-0.25 (mm)	0.25-0.075 (mm)	0.075-0.005 (mm)	0.005-0.002 (mm)	< 0.002 (mm)
RS12	538651.51	3401972.29	220-230		8.3	59.6	19.1	13.0
RS12	538651.51	3401972.29	230-240		8.7	52.6	23.7	15.0
RS13	539782.99	3401671.37	0-10		3.2	72.4	9.6	14.8
RS13	539782.99	3401671.37	10-20		3.6	72.1	7.8	16.5
RS13	539782.99	3401671.37	20-30		12.5	61.9	10.9	14.7
RS13	539782.99	3401671.37	30-40		3.8	62.8	18.2	15.2
RS13	539782.99	3401671.37	40-50		11.1	52.9	20.1	15.9
RS13	539782.99	3401671.37	50-60		7.5	57.9	18.8	15.8
RS13	539782.99	3401671.37	60-70		7.0	61.1	15.0	16.9
RS13	539782.99	3401671.37	70-80		8.8	64.3	13.7	13.2
RS13	539782.99	3401671.37	80-90		7.5	71.2	7.0	14.3
RS13	539782.99	3401671.37	90-100		12.2	51.3	20.6	15.9
RS13	539782.99	3401671.37	100-110		12.3	59.1	15.8	12.8
RS13	539782.99	3401671.37	110-120		4.4	60.9	20.6	14.1
RS13	539782.99	3401671.37	120-130		7.7	58.7	20.3	13.3
RS13	539782.99	3401671.37	130-140		4.1	73.5	10.0	12.4
RS13	539782.99	3401671.37	140-150		14.3	48.2	19.0	18.5
RS13	539782.99	3401671.37	150-160		4.3	75.3	6.8	13.6
RS13	539782.99	3401671.37	160-170		6.8	60.7	14.5	18
RS13	539782.99	3401671.37	170-180		5.6	70.2	9.9	14.3
RS13	539782.99	3401671.37	180-190		12.9	52.4	14.5	20.2
RS13	539782.99	3401671.37	190-200		3.8	66.7	9.8	19.7
RS13	539782.99	3401671.37	200-210		5.4	68.2	12.4	14.0
RS13	539782.99	3401671.37	210-220		5.9	68.6	10.6	14.9
RS13	539782.99	3401671.37	220-230		9.4	64.9	10.9	14.8
RS13	539782.99	3401671.37	230-240		4.2	74.8	8.1	12.9
RS14	541287.63	3401430.63	0-10		3.8	69.9	12.6	13.7
RS14	541287.63	3401430.63	10-20		7.2	64.9	12.6	15.3
RS14	541287.63	3401430.63	20-30		8.5	64.6	15.8	11.1
RS14	541287.63	3401430.63	30-40		5.3	68.3	14.2	12.2
RS14	541287.63	3401430.63	40-50		8.1	64.1	13.5	14.3
RS14	541287.63	3401430.63	50-60		3.7	70.5	9.4	16.4
RS14	541287.63	3401430.63	60-70		9.0	68.4	7.2	15.4
RS14	541287.63	3401430.63	70-80		10	72.7	3.3	14.0
RS14	541287.63	3401430.63	80-90		5.7	66.3	15.9	12.1
RS14	541287.63	3401430.63	90-100		12.3	65.0	6.9	15.8
RS14	541287.63	3401430.63	100-110		3.9	67.9	11.5	16.7
RS14	541287.63	3401430.63	110-120		6.9	68.4	11.3	13.4
RS14	541287.63	3401430.63	120-130		3.3	74.6	8.8	13.3

Table S1 (continued)

Samples ID	Longitude	Latitude	Depth (cm)	Particle composition (%)				
				0.50-0.25 (mm)	0.25-0.075 (mm)	0.075-0.005 (mm)	0.005-0.002 (mm)	< 0.002 (mm)
RS14	541287.63	3401430.63	130-140		6.7	71.0	4.9	17.4
RS14	541287.63	3401430.63	140-150		9.7	70.4	8.6	11.3
RS14	541287.63	3401430.63	150-160		10.9	57.5	18.9	12.7
RS14	541287.63	3401430.63	160-170		5.9	62.9	17.2	14.0
RS14	541287.63	3401430.63	170-180		8.9	68.1	11.1	11.9
RS14	541287.63	3401430.63	180-190		7.7	72.1	5.2	15.0
RS14	541287.63	3401430.63	190-200		12.4	52.9	17.6	17.1
RS14	541287.63	3401430.63	200-210		10.9	66.8	7.6	14.7
RS14	541287.63	3401430.63	210-220		5.7	69.6	7.2	17.5
RS14	541287.63	3401430.63	220-230		10.4	59.6	14	16.0
RS14	541287.63	3401430.63	230-240		11.3	63.5	8.7	16.5
RS15	531393.16	3395628.76	0-10		1.6	75.5	16.9	6.0
RS15	531393.16	3395628.76	10-20		0.3	89.7	6.9	3.1
RS15	531393.16	3395628.76	20-30		11.9	77.0	8.0	3.1
RS15	531393.16	3395628.76	30-40		4.8	81.5	7.5	6.2
RS15	531393.16	3395628.76	40-50		4.8	74.0	10.6	10.6
RS15	531393.16	3395628.76	50-60		2.8	84.9	9.2	3.1
RS15	531393.16	3395628.76	60-70		3.7	76.0	15.2	5.1
RS15	531393.16	3395628.76	70-80		11.0	62.6	10.5	15.9
RS15	531393.16	3395628.76	80-90		12.4	56.1	9.8	21.7
RS15	531393.16	3395628.76	90-100		14.7	62.3	7.4	15.6
RS15	531393.16	3395628.76	100-110		13.8	57.9	11.3	17.0
RS15	531393.16	3395628.76	110-120		11.3	58.2	10.5	20.0
RS15	531393.16	3395628.76	120-130		16.0	58.3	10.1	15.6
RS15	531393.16	3395628.76	130-140		5.8	53.8	17.2	23.2
RS15	531393.16	3395628.76	140-150		12.0	57.4	10.0	20.6
RS16	533042.24	3394376.90	0-10		13.0	52.8	11.1	23.1
RS16	533042.24	3394376.90	10-20		16.5	64.6	5.7	13.2
RS16	533042.24	3394376.90	20-30		10.1	59.5	11.0	19.4
RS16	533042.24	3394376.90	30-40	1.0	10.4	62.1	5.6	20.9
RS16	533042.24	3394376.90	40-50		9.5	62.1	11.1	17.3
RS16	533042.24	3394376.90	50-60		12.3	61.6	12.4	13.7
RS16	533042.24	3394376.90	60-70		1.2	85.2	8.8	4.8
RS16	533042.24	3394376.90	70-80		10.4	61.8	10.1	17.7
RS16	533042.24	3394376.90	80-90		1.5	84.6	8.1	5.8
RS16	533042.24	3394376.90	90-100		10.7	61.4	11.6	16.3
RS16	533042.24	3394376.90	100-110		12.8	58.9	10.2	18.1
RS16	533042.24	3394376.90	110-120	2.2	10.9	62.3	10.3	14.3
RS17	534342.24	3395532.46	0-10		18.4	59.7	11.5	10.4

Table S1 (continued)

Samples ID	Longitude	Latitude	Depth (cm)	Particle composition (%)				
				0.50-0.25 (mm)	0.25-0.075 (mm)	0.075-0.005 (mm)	0.005-0.002 (mm)	< 0.002 (mm)
RS17	534342.24	3395532.46	10-20		14.4	54.2	8.2	23.2
RS17	534342.24	3395532.46	20-30		26.1	45.3	17	11.6
RS17	534342.24	3395532.46	30-40		5.9	62.6	14.3	17.2
RS17	534342.24	3395532.46	40-50		19.1	48.5	13.8	18.6
RS17	534342.24	3395532.46	50-60		10.8	60.2	16.3	12.7
RS17	534342.24	3395532.46	60-70		38.4	42.1	11.5	8.0
RS17	534342.24	3395532.46	70-80		17.8	45.0	21.1	16.1
RS17	534342.24	3395532.46	80-90		10.6	52.7	15.8	20.9
RS17	534342.24	3395532.46	90-100	3.1	6.6	63.1	16.5	10.7
RS17	534342.24	3395532.46	100-110		20.2	47.6	19.5	12.7
RS17	534342.24	3395532.46	110-120		4.5	70.3	13.4	11.8
RS18	535774.65	3397650.99	0-10		6	66.7	11.7	15.6
RS18	535774.65	3397650.99	10-20		11.3	57.2	12.6	18.9
RS18	535774.65	3397650.99	20-30		10.7	65.0	9.9	14.4
RS18	535774.65	3397650.99	30-40		13.3	60.2	8.8	17.7
RS18	535774.65	3397650.99	40-50	27.9	25.4	18.3	13.3	15.1
RS18	535774.65	3397650.99	50-60	23.5	30.1	28.5	6.2	11.7
RS18	535774.65	3397650.99	60-70	17	25.2	41.6	6.8	9.4
RS18	535774.65	3397650.99	70-80	1.9	25.1	51.8	7.1	14.1
RS18	535774.65	3397650.99	80-90		8.7	80.8	3.7	6.8
RS18	535774.65	3397650.99	90-100		71.5	21.5	5.4	1.6
RS18	535774.65	3397650.99	100-110		8.4	64.4	9.3	17.9
RS18	535774.65	3397650.99	110-120		8.5	71.5	6.9	13.1
RS18	535774.65	3397650.99	120-130		9.2	63.0	10.7	17.1
RS18	535774.65	3397650.99	130-140		12.5	59.5	13.6	14.4
RS18	535774.65	3397650.99	140-150	1.5	12.5	58.0	10.6	17.4
RS18	535774.65	3397650.99	150-160		15.4	58.2	10.5	15.9
RS18	535774.65	3397650.99	160-170		9.9	59.0	10.6	20.5
RS18	535774.65	3397650.99	170-180		11.2	67.2	6.2	15.4
RS18	535774.65	3397650.99	180-190		9.8	63.8	9.1	17.3
RS18	535774.65	3397650.99	190-200		4.3	70.2	10.7	14.8
RS18	535774.65	3397650.99	200-210		2.2	76.7	5.4	15.7
RS18	535774.65	3397650.99	210-220		11.5	58.9	12.1	17.5
RS18	535774.65	3397650.99	220-230		11.1	64.6	8.1	16.2
RS18	535774.65	3397650.99	230-240		11.1	66.5	9.2	13.2
RS19	536785.76	3396663.95	0-10		11.4	71	5.2	12.4
RS19	536785.76	3396663.95	10-20		15.2	67.7	3.8	13.3
RS19	536785.76	3396663.95	20-30		9.6	47.0	24.8	18.6
RS19	536785.76	3396663.95	30-40		10.9	55.7	17.7	15.7

Table S1 (continued)

Samples ID	Longitude	Latitude	Depth (cm)	Particle composition (%)				
				0.50-0.25 (mm)	0.25-0.075 (mm)	0.075-0.005 (mm)	0.005-0.002 (mm)	< 0.002 (mm)
RS19	536785.76	3396663.95	40-50		10	44.5	26.8	18.7
RS19	536785.76	3396663.95	50-60		6.4	58.1	15.7	19.8
RS19	536785.76	3396663.95	60-70		13.8	53.0	21.5	11.7
RS19	536785.76	3396663.95	70-80		11.2	51.4	24.5	12.9
RS19	536785.76	3396663.95	80-90	5	10.6	52	19.1	13.3
RS19	536785.76	3396663.95	90-100		11.1	58.7	14.1	16.1
RS19	536785.76	3396663.95	100-110		13.1	54.8	8.8	23.3
RS19	536785.76	3396663.95	110-120		11.3	55.1	12.1	21.5
RS19	536785.76	3396663.95	120-130		4.3	48.2	19.0	28.5
RS19	536785.76	3396663.95	130-140		8.8	51.4	20.8	19
RS19	536785.76	3396663.95	140-150		21.9	39.9	16.3	21.9
RS19	536785.76	3396663.95	150-160	6.8	10.1	45.7	21.3	16.1
RS19	536785.76	3396663.95	160-170		0.8	52.7	22.2	24.3
RS19	536785.76	3396663.95	170-180		9.2	49.1	16.4	25.3
RS19	536785.76	3396663.95	180-190		5.3	46.8	28.4	19.5
RS19	536785.76	3396663.95	190-200		10.3	43.7	14.3	31.7
RS19	536785.76	3396663.95	200-210		5.4	51.4	22.1	21.1
RS19	536785.76	3396663.95	210-220		11.4	34.4	28.9	25.3
RS20	535582.06	3399998.22	0-10		24.6	49.3	12.3	13.8
RS20	535582.06	3399998.22	10-20		7.8	45.6	23.4	23.2
RS20	535582.06	3399998.22	20-30	4.1	21.4	51.0	10.7	12.8
RS20	535582.06	3399998.22	30-40	3.6	7.8	48.9	21.5	18.2
RS20	535582.06	3399998.22	40-50		22.2	42.1	23.2	12.5
RS20	535582.06	3399998.22	50-60		10.1	57.3	19.1	13.5
RS20	535582.06	3399998.22	60-70		5.2	53.1	20.2	21.5
RS20	535582.06	3399998.22	70-80		19.8	48.5	18.4	13.3
RS20	535582.06	3399998.22	80-90		14.5	55.7	16.1	13.7
RS20	535582.06	3399998.22	90-100		29.9	36.1	18.0	16
RS20	535582.06	3399998.22	100-110		16.7	44.6	22.0	16.7
RS20	535582.06	3399998.22	110-120		10.5	52.3	19.1	18.1
RS20	535582.06	3399998.22	120-130		21.0	50.0	16.6	12.4
RS21	537556.14	3398180.62	0-10		17.7	53.6	15.2	13.5
RS21	537556.14	3398180.62	10-20		18.0	50.7	18.5	12.8
RS21	537556.14	3398180.62	20-30		5.6	58.9	18.7	16.8
RS21	537556.14	3398180.62	30-40		6.5	56.4	26.5	10.6
RS21	537556.14	3398180.62	40-50		17.0	50.3	15.3	17.4
RS21	537556.14	3398180.62	50-60		21.4	48.0	13.6	17
RS21	537556.14	3398180.62	60-70		23.8	37.6	9.4	29.2
RS21	537556.14	3398180.62	70-80	3.9	8.8	58.1	15.1	14.1

Table S1 (continued)

Samples ID	Longitude	Latitude	Depth (cm)	Particle composition (%)				
				0.50-0.25 (mm)	0.25-0.075 (mm)	0.075-0.005 (mm)	0.005-0.002 (mm)	< 0.002 (mm)
RS21	537556.14	3398180.62	80-90		9.4	53.5	22	15.1
RS21	537556.14	3398180.62	90-100		19.3	42.8	21.1	16.8
RS21	537556.14	3398180.62	100-110		25.6	39.6	14.7	20.1
RS21	537556.14	3398180.62	110-120		14.5	47.7	17.0	20.8
RS21	537556.14	3398180.62	120-130		11.7	53.1	21.4	13.8
RS21	537556.14	3398180.62	130-140		24.2	47.6	14.3	13.9
RS21	537556.14	3398180.62	140-150		7.4	55.6	22.5	14.5
RS21	537556.14	3398180.62	150-160		13.0	60.8	14.9	11.3
RS22	536051.50	3401021.37	0-10		18.4	56.9	13.4	11.3
RS22	536051.50	3401021.37	10-20		32.9	44.9	11.8	10.4
RS22	536051.50	3401021.37	20-30		13.9	41.2	26.8	18.1
RS22	536051.50	3401021.37	30-40		26.4	44.7	18.0	10.9
RS22	536051.50	3401021.37	40-50		34.9	43.1	13.8	8.2
RS22	536051.50	3401021.37	50-60		12.9	53.8	17.9	15.4
RS22	536051.50	3401021.37	60-70		5.8	59.2	20.7	14.3
RS22	536051.50	3401021.37	70-80		9.2	53.0	21.1	16.7
RS22	536051.50	3401021.37	80-90	9.9	23.4	38.7	18.7	9.3
RS22	536051.50	3401021.37	90-100		22.7	48.2	16.7	12.4
RS22	536051.50	3401021.37	100-110		16.9	47.8	15.9	19.4
RS22	536051.50	3401021.37	110-120		7.9	55.5	18.9	17.7
RS23	537291.32	3400949.14	0-10		8.8	61.6	15.2	14.4
RS23	537291.32	3400949.14	10-20		8.2	55.5	22.3	14.0
RS23	537291.32	3400949.14	20-30		33.2	42	13.4	11.4
RS23	537291.32	3400949.14	30-40		8.2	63.5	10.6	17.7
RS23	537291.32	3400949.14	40-50		18	49.2	22.2	10.6
RS23	537291.32	3400949.14	50-60		9.4	57.0	21.7	11.9
RS23	537291.32	3400949.14	60-70		28.9	45.6	14.7	10.8
RS23	537291.32	3400949.14	70-80		10.6	62.6	11.7	15.1
RS23	537291.32	3400949.14	80-90		31.6	44.6	15.3	8.5
RS23	537291.32	3400949.14	90-100		19.3	50.5	14.4	15.8
RS23	537291.32	3400949.14	100-110		17.6	39.1	33.2	10.1
RS23	537291.32	3400949.14	110-120		17.6	61.0	10.1	11.3
RS24	538001.51	3399697.29	0-10		10.5	54.0	25.6	9.9
RS24	538001.51	3399697.29	10-20	4.0	4.1	62.5	16.3	13.1
RS24	538001.51	3399697.29	20-30		26.1	50.0	10.5	13.4
RS24	538001.51	3399697.29	30-40		15.1	55.6	16.3	13
RS24	538001.51	3399697.29	40-50		8.2	63.5	9.7	18.6
RS24	538001.51	3399697.29	50-60	4.7	25.0	47.5	13.3	9.5
RS24	538001.51	3399697.29	60-70		20.1	51.4	15.5	13.0

Table S1 (continued)

Samples ID	Longitude	Latitude	Depth (cm)	Particle composition (%)				
				0.50-0.25 (mm)	0.25-0.075 (mm)	0.075-0.005 (mm)	0.005-0.002 (mm)	< 0.002 (mm)
RS24	538001.51	3399697.29	70-80	4.3	22.4	50.3	11.6	11.4
RS24	538001.51	3399697.29	80-90		4.5	54.2	22.5	18.8
RS24	538001.51	3399697.29	90-100		19.3	48.7	14.7	17.3
RS24	538001.51	3399697.29	100-110		20.4	45.9	17.6	16.1
RS24	538001.51	3399697.29	110-120		19.3	51.9	16.5	12.3
RS24	538001.51	3399697.29	120-130		5.8	60.8	18.8	14.6
RS25	540505.22	3399528.77	0-10		19.8	46.8	18.9	14.5
RS25	540505.22	3399528.77	10-20		11	42.3	28.2	18.5
RS25	540505.22	3399528.77	20-30		26.2	49.1	16.4	8.3
RS25	540505.22	3399528.77	30-40		13.9	57.8	14.8	13.5
RS25	540505.22	3399528.77	40-50		22.8	45.4	16.9	14.9
RS25	540505.22	3399528.77	50-60		5.0	59.3	20.8	14.9
RS25	540505.22	3399528.77	60-70		31.3	37.3	17.2	14.2
RS25	540505.22	3399528.77	70-80		10.7	50.7	20.6	18
RS25	540505.22	3399528.77	80-90		25.6	45.9	11.6	16.9
RS25	540505.22	3399528.77	90-100		11.1	59.3	17.5	12.1
RS25	540505.22	3399528.77	100-110		22.5	47.0	16.1	14.4
RS25	540505.22	3399528.77	110-120		8.4	57.7	21.9	12
RS25	540505.22	3399528.77	120-130		12.5	54.2	21.9	11.4
RS25	540505.22	3399528.77	130-140		23.5	40.1	20.0	16.4
RS25	540505.22	3399528.77	140-150		13.7	49.5	18.9	17.9
RS25	540505.22	3399528.77	150-160		7.5	64.8	17.8	9.9
RS25	540505.22	3399528.77	160-170		11.5	60.7	11.6	16.2
RS25	540505.22	3399528.77	170-180		7.3	67.6	10.4	14.7
RS25	540505.22	3399528.77	180-190		6.4	76.1	8.4	9.1
RS25	540505.22	3399528.77	190-200		8.8	66.0	9.5	15.7
RS25	540505.22	3399528.77	200-210		8.8	57.2	13.9	20.1
RS26	538290.40	3401129.70	0-10		1.9	55.5	22.6	20
RS26	538290.40	3401129.70	10-20		8.6	55.6	21.2	14.6
RS26	538290.40	3401129.70	20-30		11.8	64.1	8.0	16.1
RS26	538290.40	3401129.70	30-40		11.8	58.3	13.7	16.2
RS26	538290.40	3401129.70	40-50		14.5	59.4	10.1	16.0
RS26	538290.40	3401129.70	50-60		11.9	64.5	3.0	20.6
RS26	538290.40	3401129.70	60-70		5.9	74.6	2.9	16.6
RS26	538290.40	3401129.70	70-80		7.2	72.2	5.4	15.2
RS26	538290.40	3401129.70	80-90		2.7	65.3	11.8	20.2
RS26	538290.40	3401129.70	90-100		11.3	59.2	16.3	13.2
RS26	538290.40	3401129.70	100-110		3.5	72.1	8.8	15.6
RS26	538290.40	3401129.70	110-120		12.3	62.1	11.3	14.3

Table S1 (continued)

Samples ID	Longitude	Latitude	Depth (cm)	Particle composition (%)				
				0.50-0.25 (mm)	0.25-0.075 (mm)	0.075-0.005 (mm)	0.005-0.002 (mm)	< 0.002 (mm)
RS26	538290.40	3401129.70	120-130		8.9	68.0	6.8	16.3
RS26	538290.40	3401129.70	130-140		8.5	66.1	11	14.4
RS26	538290.40	3401129.70	140-150		10.5	59.7	15.2	14.6
RS26	538290.40	3401129.70	150-160		6.8	68.2	12.1	12.9
RS26	538290.40	3401129.70	160-170		13.0	66.2	7.9	12.9
RS26	538290.40	3401129.70	170-180		5.6	73.1	10.4	10.9
RS27	540047.81	3400888.96	0-10		4.1	65.4	19.0	11.5
RS27	540047.81	3400888.96	10-20		10.1	59.5	16.7	13.7
RS27	540047.81	3400888.96	20-30		4.7	59.5	19.3	16.5
RS27	540047.81	3400888.96	30-40		14.2	54.3	16.8	14.7
RS27	540047.81	3400888.96	40-50		6.1	68.8	10.6	14.5
RS27	540047.81	3400888.96	50-60		5.1	65.7	12.2	17.0
RS27	540047.81	3400888.96	60-70		7.8	64.7	9.2	18.3
RS27	540047.81	3400888.96	70-80		14.9	57.7	9.3	18.1
RS27	540047.81	3400888.96	80-90		4.6	60.7	19.5	15.2
RS27	540047.81	3400888.96	90-100		6.9	61.8	15.4	15.9
RS27	540047.81	3400888.96	100-110		4.5	74.9	4.4	16.2
RS27	540047.81	3400888.96	110-120		10.2	69.1	7.4	13.3
RS27	540047.81	3400888.96	120-130		6.0	73.1	10.2	10.7
RS27	540047.81	3400888.96	130-140		13.1	58.9	15.5	12.5
RS27	540047.81	3400888.96	140-150		6.2	61.1	19.6	13.1
RS28	541420.03	3400527.85	0-10		6.9	69.6	8.8	14.7
RS28	541420.03	3400527.85	10-20		4.2	67.3	8.1	20.4
RS28	541420.03	3400527.85	20-30		4.3	74.5	5.3	15.9
RS28	541420.03	3400527.85	30-40		4.7	71.1	11.0	13.2
RS28	541420.03	3400527.85	40-50		11.6	50.3	23.4	14.7
RS28	541420.03	3400527.85	50-60		13	62.6	6.4	18.0
RS28	541420.03	3400527.85	60-70		11.4	67.3	4.2	17.1
RS28	541420.03	3400527.85	70-80		3.2	61.6	17.9	17.3
RS28	541420.03	3400527.85	80-90		6.8	67.2	13.9	12.1
RS28	541420.03	3400527.85	90-100		4.1	66.8	15	14.1
RS28	541420.03	3400527.85	100-110		10.9	63.2	12.5	13.4
RS28	541420.03	3400527.85	110-120		5.9	60.4	16.0	17.7
RS28	541420.03	3400527.85	120-130		8.5	72.4	5.4	13.7
RS28	541420.03	3400527.85	130-140		11.9	61.6	12.2	14.3
RS28	541420.03	3400527.85	140-150		5.1	52.6	20.2	22.1
