# Supplementary material for: Spatial variability of soil texture fractal dimension and its application in sponge city construction
Source: PLoS One. 2026 Sep 21;21(9):e0357100. doi: 10.1371/journal.pone.0357100 (PMC13592644; doi:10.1371/journal.pone.0357100)
Supplement: Supplementary file 2 [file pone.0357100.s002.docx]

**Manuscript ID: PONE-D-25-60225**

**Response to Reviewers**

Dear Editor,

Thank you for giving us the opportunity to submit a revised draft of the manuscript “Spatial variability of soil textural fractal and its application in the construction of sponge city” for publication in the *Plos One*. We appreciate the time and effort that you and the reviewers dedicated to providing feedback on our manuscript and are grateful for the insightful comments on and valuable improvements to our paper. We have incorporated all of the suggestions made by the reviewers. Those changes are highlighted within the manuscript. Please see below, in blue, for a point-by-point response to the reviewers’ comments and concerns. All page numbers refer to the revised manuscript file with tracked changes:

**Reviewers' Comments to the Authors:**

**Reviewer 1**

This study explored effectivity approaches for characterizing the spatial variability of soll textures and determining the sol hydraulicparameters in the sponge cties by using fractal theory and geostatistics. Howeve, the relevant research methods are simple, and the application insponge city construction is insufficient. Therefore, I do not recommend this paper to be published. Major Comments:

**Author response:** We sincerely appreciate the reviewer for the critical and constructive evaluation of our manuscript, as well as the professional insights into the methodological rigor and engineering application value of our study. We fully recognize the concerns raised, and have conducted comprehensive and in-depth revisions to the manuscript, including systematic supplementation of methodological validation, detailed expansion of the engineering application in sponge city construction, and full optimization of the manuscript structure and content in accordance with all subsequent major and specific comments. We believe the revised manuscript has been significantly improved in both scientific rigor and practical application value, and has fully addressed all the concerns raised by the reviewer. Our detailed responses to the core comments are as follows:

1. The part of "Introduction" stll needs to be improved, the authors should further cariy the novelty of this work in the Introduction.

**Author response:** We sincerely appreciate the reviewers’ valuable comments. In response, we have undertaken a revision and refinement of the Introduction. First, we have expanded the literature review to incorporate the latest advances in domestic and international research on the relationship between soil fractal dimensions and hydraulic parameters, and we have articulated the existing research gaps. These include the insufficient attention to the vertical variability of soil fractal dimensions in urban sponge-city contexts, the limited application of fractal models throughout the full planning and design cycle of sponge cities, and the lack of three-dimensional spatial characterization of soil fractal features across large urban regions. Building on this foundation, we elaborate on the three core innovations of this study: a technical framework for full-profile, three-dimensional characterization of soil fractal features at the scale of large sponge-city regions; a rapid and low-cost method for deriving soil hydraulic parameters based on fractal modeling; and site-specific low-impact development (LID) facility layout strategies directly informed by soil fractal indicators. We have also optimized the logical structure of the Introduction, establishing a coherent narrative that progresses from practical engineering needs to scientific bottlenecks, research gaps, and our key innovations, thereby ensuring that the novelty of this work is clearly and prominently conveyed (**pp. 2–4, Section 1, Introduction**).

2. Does the small number of sampling points and large spatial distance meet the requirements of geostatistical spatial analysis?

**Author response:** We sincerely appreciate the reviewer’s professional question on the rationality of our sampling design for geostatistical analysis. The 28 sampling points in this study were uniformly distributed across the entire 50 km² study area, with full consideration of the representativeness of different geomorphological units, land-use types and sedimentary environments in the region, which conforms to the sampling specifications for geostatistical analysis of plain Quaternary sedimentary strata. Meanwhile, the semi-variogram fitting results show that most soil layers have a fitting determination coefficient R² > 0.7, and the nugget-to-sill ratio of most layers is below 25%, which confirms that our sampling points can effectively capture the spatial structural characteristics of soil fractal dimensions, and fully meet the accuracy requirements of geostatistical spatial analysis.

3. Please explain why 28 soil samples were collected, but only the soil water characteristics curves of 14 soil samples were analyzed.

**Author response:** We sincerely appreciate the reviewer’s question on the selection of soil samples for SWCC analysis. The 28 sampling profiles were uniformly distributed across the study area to fully characterize the spatial and vertical variability of soil fractal dimensions for 3D spatial modeling. We selected 14 representative profiles for SWCC analysis, which fully cover all soil texture types (silt, silty clay, clay), the full range of fractal dimension D values, and all typical land-use types and sedimentary units in the study area. This representative sampling strategy ensures the established fractal SWCC prediction model is robust and universal, and the verification results (R^2^>0.9 for most samples) confirm that the selected 14 samples can fully support the hydraulic parameter prediction for the entire study area.

4. Please separate "Results" and "discussion" into two parts. Some of the content in the discussion section of this paper can be moved to the resultssection. The discussion section should provide a clearer interpretation of the results.

**Author response:** We sincerely appreciate the reviewer’s suggestion regarding the manuscript structure. Following this comment, we have fully separated the Results and Discussion into two independent sections, relocated all descriptive findings from the original Discussion to the appropriate subsections of the Results. The revised Discussion now concentrates on interpreting the results, analyzing underlying mechanisms, assessing engineering implications for sponge‑city construction, and comparing our findings with existing studies. All revisions are highlighted with track changes in the updated manuscript (Results: pp. 8–20; Discussion: pp. 21–25).

5. Please add some current state of research both domestically and intemationally about the relationship between soil fractal dimension and hydrauicparameters to the introduction section. The discussion should be strengthened, as the application of spatial heterogeneity of fractal features in sponge cties has not been thoroughlydiscussed.

**Author response:** We sincerely appreciate the reviewer’s suggestion. We have completed the corresponding revisions as follows: First, we have supplemented the latest domestic and international research progress on the relationship between soil fractal dimension and hydraulic parameters in the Introduction section, and sorted out the limitations of existing studies. Second, we have strengthened the Discussion section, systematically elaborated the application of spatial heterogeneity of soil fractal features in sponge city facility selection, spatial layout and vertical design, supplemented specific engineering application scenarios, and completed the in-depth interpretation of the results. All revisions are marked with track changes in the revised manuscript.

Specific Comments:

1.Please ensure all references are up-to-date and relevant to the current study.

**Author response:** We sincerely appreciate the reviewer’s suggestion. We have comprehensively checked and revised all references in the manuscript: we updated the latest domestic and international relevant studies closely related to soil fractal dimension, soil hydraulic parameters, geostatistical analysis and sponge city construction, removed outdated and less relevant references, and standardized the citation format in strict accordance with the journal’s requirements. All revisions are marked with track changes in the revised manuscript.

2. Please improve the image quality, for example, Figure 4 is blurry.

**Author response:** We sincerely appreciate the reviewer’s suggestion. We have comprehensively improved the resolution and clarity of all figures in the manuscript, especially the blurry Figure 4. All revised high-quality figures have been updated in the resubmitted manuscript.

3. Please use italics for the variables in the formula.

**Author response:** We sincerely appreciate the reviewer’s suggestion. We have standardized the format of all formulas in the manuscript, and used italics for all variables in the formulas as required. All revisions are marked with track changes in the revised manuscript.

4. Please standardize the number of decimal places retained in Table 1.

**Author response:** We sincerely appreciate the reviewer’s suggestion. We have standardized the number of decimal places retained for all data in Table 1, and the relevant revisions are marked with track changes in the revised manuscript.

5. Please add some summary of the results in the conclusion section.

**Author response:** We sincerely appreciate the reviewer’s suggestion. We have supplemented a summary of the core research results in the Conclusion section, including the distribution range of soil fractal dimensions, the prediction accuracy of the fractal model for soil water characteristic curves, and the differentiated sponge city construction strategies based on fractal features. All revisions are marked with track changes in the revised manuscript.

6. Please modify the citation format of the references in the paper according to the requirements of the journal.

**Author response:** We have strictly modified the citation format of in-text references and the list of references at the end of the paper in full accordance with the journal’s requirements. All revisions are marked with track changes in the revised manuscript.

**Reviewer 2**

Suggestion 1. It is argued in section 4.1, "Soil fractal dimensions become more uniform with increasing depth across the region. Thismay cause by the spatial variability of the regional lithology and artificial fill."The authors are suggested to reconsider the logic of the phrase:

**Author response:** We sincerely appreciate the reviewer’s suggestion. We have re-examined and revised the logic of this phrase, corrected the grammatical error, and updated the sentence to: "Soil fractal dimensions become more uniform with increasing depth across the region. This trend is mainly controlled by the stable regional lithology in deep layers, while the heterogeneity of shallow soils is strongly influenced by artificial fill." The revision is marked with track changes in the revised manuscript (Page 22, Section 4.1).

Suggestion 2. The Abstract daims that, the essay attempts to demonstrate "that fractal theory provides an eficient approach to characterize thepatial distribution of sol PSD and estabish the relationship between sol D values and hydrauic parameters, ofering a scientific basis for planningand construction of sponge city not only in Chian, but also in similar regions worldwide.

**Author response:** We sincerely appreciate the reviewer’s suggestion. In response, we have corrected the spelling error in the Abstract, revised the overstated statements, incorporated relevant literature on soil investigations in Wuhan and on the relationship between soil D values and hydraulic parameters, and clearly defined both the innovation and the applicable scope of our method while avoiding exaggerated expressions. All revisions are marked with track changes in the Abstract of the revised manuscript.

Suggestion 2. The Abstract daims that, the essay attempts to demonstrate "that fractal theory provides an eficient approach to characterize thepatial distribution of sol PSD and estabish the relationship between sol D values and hydrauic parameters, ofering a scientific basis for planningand construction of sponge city not only in Chian, but also in similar regions worldwide.

**Author response:** We sincerely appreciate the reviewer’s suggestion. In response, we have supplemented and reorganized the relevant literature on soil investigations in Wuhan and on the relationship between soil D values and hydraulic parameters, and clarified that existing studies primarily focus on non‑urban settings and shallow soil layers, lacking full‑profile 3D fractal characterization for sponge‑city construction areas in Wuhan. We have also revised overstated descriptions throughout the manuscript and precisely articulated the innovation of this study: the development of a 3D full‑profile spatial model of soil fractal dimensions for Wuhan’s sponge‑city demonstration area, and the proposal of a targeted sponge‑facility layout method informed by fractal features. These contributions address a key local research gap and provide direct technical support for sponge‑city implementation. All revisions are marked with track changes in the updated manuscript.

**Reviewer 3**

Suggestion 1. It is argued in section 4.1, "Soil fractal dimensions become more uniform with increasing depth across the region. Thismay cause by the spatial variability of the regional lithology and artificial fill."The authors are suggested to reconsider the logic of the phrase:

**Author response:** We sincerely appreciate the reviewer’s positive evaluation and constructive suggestions. Accordingly, we have made the following revisions. First, we supplemented the applicable conditions of our method for similar Quaternary sedimentary plain regions worldwide, refined the previously overgeneralized statements regarding global applicability, and added supporting evidence from studies in comparable areas to clarify the method’s replicability and transferability. Second, we incorporated a systematic comparison between our approach and conventional methods (laboratory pressure plate tests and field test‑pit measurements), clearly demonstrating the substantial advantages of our method in data‑collection efficiency, cost control, and spatial continuity for large‑scale urban planning, as well as its contribution to enhancing sponge‑city planning. All revisions are marked with track changes in the updated manuscript.
