# Supplementary material for: Spatial variability of soil texture fractal dimension and its application in sponge city construction
Source: PLoS One. 2026 Sep 21;21(9):e0357100. doi: 10.1371/journal.pone.0357100 (PMC13592644; doi:10.1371/journal.pone.0357100)
Supplement: Supplementary file 3 [file pone.0357100.s003.docx]

**Manuscript ID: PONE-D-26-14596**

**Manuscript Title: Spatial variability of soil texture fractal dimension and its application in sponge city construction**

**Corresponding Author: Yun Li**

We thank the editors and two anonymous reviewers for their valuable and constructive comments, which have greatly helped improve the quality of our manuscript. Every critical suggestion has been carefully reviewed and fully addressed in the revision. We have systematically revised the entire manuscript, and specifically added more in-depth discussion and extended the analysis of the research implications in the Discussion section as suggested. All revisions are marked with red track changes for easy identification. Detailed point-by-point responses to the comments from Reviewer #1 and Reviewer #2 are presented below.

**Response to Reviewer 1#:**

**Comments.**

The authors have fully and effectively addressed all questions raised by the reviewers. This manuscript meets the criteria for publication, and I recommend its acceptance for publication.

**Author response:**

We appreciate your positive evaluation of our revised manuscript.

**Response to Reviewer 2#:**

**Comment 1.**

I recommend acceptance after major revisions. The manuscript addresses an interesting and relevant topic and is generally well written, scientifically structured, and supported by a good-quality dataset and appropriate data analysis. The study is promising and has the potential to make a valuable contribution to the field. However, the manuscript would benefit a lot from a more in-depth discussion of the results, clearer presentation of their practical implications, and stronger integration with the existing international literature. You have to incorporate additional references to better position the findings within the current state of knowledge and to demonstrate how this study advances the field. The specific comments below are intended to strengthen the manuscript and improve its scientific impact.

**Author response:**

Thank you very much for your valuable and constructive comments. We have revised the manuscript as you suggested.

**Comment 2.**

Line 48: Please provide the publication year of Mandelbrot's work.

**Author response:**

We have clearly marked the publication year 1977 in reference [15] for Mandelbrot’s work. The complete citation is: [15] Mandelbrot BB. Fractals: from, chance, and dimension. San Francisco: WH Freeman; 1977.

**Comment 3.**

Lines 111–114: The complete USDA textural classes (e.g., silty clay loam, clay loam, sandy clay loam, silt loam, etc.) are not presented. Given the high heterogeneity of the study area, including the full textural classification would be important.

**Author response:**

This study is oriented toward the practical engineering application of sponge city construction. The particle size classification adopted follows the Standard for Engineering Classification of Soil (Chinese national standard), which is universally used in domestic hydrogeological surveys and municipal engineering design. This classification system is directly compatible with the technical specifications of local sponge city construction, ensuring that our research conclusions can be directly applied to engineering practice. According to Standard for Engineering Classification of Soil, the dominant soil textures classes are clarified silt, silty clay and clay in the study area. The revised content has been updated in the manuscript. (Lines 125-128, new MS)

**Comment 4.**

Lines 141–142: Please provide supporting references.

**Author response:**

We have added supporting references [27,28] at lines 155–156 (lines 141–142, old MS) in the revised manuscript. The complete citations are: [27] Wang JM, Lu X, Feng Y, Yang RX. Integrating multi-fractal theory and geo-statistics method to characterize the spatial variability of particle size distribution of minesoils. Geoderma, 2018; 317: 39-46. [28] Wang JM, Zhang JR, Feng Y. Characterizing the spatial variability of soil particle size distribution in an underground coal mining area: An approach combining multi-fractal theory and geostatistics. Catena 2019; 176, 94-103.

**Comment 5.**

Lines 154–159: This section could be shortened, as it excessively describes information already presented in the table.

**Author response:**

We have shortened this section and removed redundant details already presented in the table. (Lines 168-171, new MS)

**Comment 6.**

Line 171: The statement “which indicates a more compact soil structure at greater depths” is not fully supported, since the mean values are practically similar throughout the profile (0–240 cm).

**Author response:**

We have revised this statement. The original inference about compact soil structure was not sufficiently supported by the data. The revised text only describes the narrower variation range and higher homogeneity of D values at greater depths, without overinterpreting soil structural characteristics. (Lines 182-183, new MS)

**Comment 7.**

Lines 173–174: This information is repetitive and has already been mentioned in the previous paragraph. Avoid repeating obvious descriptions of the results.

**Author response:**

We have shortened this section and removed the repetitive descriptions already stated in the previous paragraph. (Lines 184-185, new MS)

**Comment 8.**

Lines 202–216: What conclusion can be drawn from this description of the results? The discussion lacks coherence and a clear interpretation of the findings.

**Author response:**

We have revised this section. We added a clear concluding interpretation of the spatial distribution pattern, sorted out the logical link between D value characteristics, formation causes and soil particle composition, and improved the coherence of result description and interpretation. (Lines 213-229, new MS)

**Comment 9.**

Line 230: Please reformulate this sentence for clarity.

**Author response:**

We have reformulated this sentence. We corrected the grammatical error and clarified the logical relationship between the influencing factors and the depth-dependent uniformity of D values. (Line 243-246, new MS)

**Comment 10&11.**

Lines 245–249: Where does the fractal dimension parameter appear in the model? How was matrix suction calculated? This section requires a more detailed explanation.

Line 250: The statement “air-entry values were assigned based on empirical data for different soil textures” requires clarification. How were these values assigned? This information should be explained. Table 4 presents only four values, and their origin is unclear.

**Author response:**

We appreciate the reviewer’s insightful question. The fractal dimension parameter appears in the model through its empirical and theoretical linkage to the van Genuchten (VG) soil water characteristic curve (SWCC) parameters. As detailed in the new revised manuscript, we employed the Bird et al. (2000) and Huang et al. (2002) fractal model related D to predict the SWCCs. (Lines 256-270)

We have clarified the assignment rule and origin of the air-entry values. We explained in the text that the values are matched to soil texture classes and sourced from a classic empirical dataset, with a supporting reference [37] added. (Lines 273-280)

**Comment 12&13.**

Line 254: The sentence “For instance, sample RS04 exhibited the highest water retention capacity, while RS01 showed the lowest” is unnecessary. The authors should interpret the results rather than describe them so simply.

Lines 255–256: In what way are the results consistent with previous findings? How was this convergence observed? Please provide a clearer explanation.

**Author response:**

We have revised this section to address both comments.

For Line 254: We removed the simple descriptive statement of individual samples, and replaced it with an interpretive explanation of the pore-scale mechanism linking fractal dimension to water retention capacity. (Lines 283-289, new MS)

For Lines 255–256: We clarified that the consistency with previous findings lies in the positive correlation between fractal dimension and water retention performance, which matches the established conclusion in fractal soil hydrology research. Corresponding supporting references [11,38] have been added. (Lines 287-287, new MS)

**Comment 14.**

Lines 265–269: The argumentation needs to be better supported with solid references and a more objective discussion rather than a purely qualitative interpretation.

**Author response:**

We have revised this section. We supplemented additional references to support the relationship between soil texture and hydraulic properties, linked the engineering implication to the study area’s climatic characteristics with regional research evidence, and added notes on other influencing factors to make the discussion more objective and evidence-based. (Lines 297-307, new MS)

**Comment 15.**

Lines 274–275: The statement is too generic. It remains unclear how the results can be applied in practical urban management and planning.

**Author response:**

We have revised this generic statement. We clarified the specific practical application paths of the results, including hydrological functional zoning, targeted sponge facility layout, and optimization of geological survey workload in urban planning and management, to make the practical application value of the findings more explicit. (Lines 313-322, new MS)

**Comment 16.**

Lines 276–281: This section is largely descriptive and somewhat repetitive of the Results section. The discussion should move beyond description and focus on interpreting the findings and addressing the underlying research problem.

**Author response:**

We have revised this section. We removed the descriptive content that repeated the Results section, and shifted the focus to interpretation: we explained the genetic mechanism behind the vertical differentiation of fractal dimensions, clarified the advantage of fractal dimension as a comprehensive indicator over traditional grain-size parameters, and discussed the practical value of this finding for optimizing hydrological model parameterization and improving simulation accuracy in sponge city planning. (Lines 323-327, new MS)

**Comment 17.**

Lines 286–287: What would this sampling strategy be used for? The conclusion that geological and natural factors control the spatial distribution of soil texture is rather obvious and cannot be considered a major finding of the study. What are the real implications of your results?

**Author response:**

We have reorganized this section of the discussion. We removed the specific descriptions of sponge facility layout that overlap with Section 4.3, and shifted the focus to the core contribution of this study: the fractal-based method provides an efficient solution for obtaining deep soil hydraulic parameters at the regional scale, addressing a common limitation of traditional measurement methods. The revised content is more concise, avoids repetition with subsequent sections, and logically leads to the detailed practical implications in Section 4.3. (Lines 331-337, new MS)

**Comment 18.**

In the subsequent sections, particularly Section 4.3, the practical implications are discussed in greater detail. This final part of the discussion could be better organized to be more direct, less repetitive, and more focused on the study’s actual contributions.

**Author response:**

We sincerely appreciate the reviewer’s constructive suggestion regarding the practical implications in Section 4.3. We agree that the previous discussion was somewhat diluted by redundant background information and lacked a sharp focus on actionable outcomes. To address this, we have thoroughly revised the section (titled “Optimization of Sponge City Facility Design Based on Fractal Characteristics” in the new MS) by removing repetitive descriptions of general sponge city principles and reorienting the narrative toward specific, data-driven insights. The revised content explicitly translates our findings into engineering guidelines—for instance, specifying that soil fractal dimensions (D) between 2.60 and 2.75 require 15–20 cm crushed stone layers to prevent clogging, and that depression storage depths of 30–50 cm optimize retention in high-D zones. Furthermore, we have strengthened the discussion on spatial decision-making by mapping these quantitative thresholds to local contexts, such as recommending permeable pavements for the Xingfu and Zhujiace communities (D<2.65) and engineered rain gardens for the Fu River alluvial plains (D>2.75). These refinements ensure the discussion is concise, targeted, and provides a robust, quantitative foundation for optimizing sponge city designs in analogous hydrogeological settings. (Line 358-420, new MS)

**Comment 19.**

Overall, the findings are interesting but the discussion should be more objective and should better compare the findings with studies conducted elsewhere in the world. The authors need to demonstrate more clearly how their work advances scientific knowledge and why the proposed approach is promising for practical applications.

**Author response:**

We have revised the discussion section. We added comparisons with international studies to place our findings in the global research context, and clarified that this study extends fractal analysis from shallow topsoil to deep urban vadose zones, advancing the application scope of existing methods. We also explained the practical advantages of the fractal-based approach in survey efficiency and cost, as well as its generalizability to similar regions. The revised discussion is more objective, with more explicit scientific contributions and application prospects. (Lines 357-420, new MS)
