# Supplementary material for: Spatial variability of soil texture fractal dimension and its application in sponge city construction
Source: PLoS One. 2026 Sep 21;21(9):e0357100. doi: 10.1371/journal.pone.0357100 (PMC13592644; doi:10.1371/journal.pone.0357100)
Supplement: Peer review history

# Author comment: Author response to Decision Letter 0

**DOI:** 10.1371/journal.pone.0357100.r001

25 Mar 2026

Attachment Submitted filename: Response to Reviewers-PONE-D-25-60225.docx

# Peer review: Decision Letter 0

**DOI:** 10.1371/journal.pone.0357100.r002

**Authors:** Bappa Mukherjee

16 Jun 2026

Dear Dr. Li,

Thank you for submitting your manuscript to PLOS ONE. After careful consideration, we feel that it has merit but does not fully meet PLOS ONE’s publication criteria as it currently stands. Therefore, we invite you to submit a revised version of the manuscript that addresses the points raised during the review process.

Please submit your revised manuscript by Jul 31 2026 11:59PM. If you will need more time than this to complete your revisions, please reply to this message or contact the journal office at plosone@plos.org. When you're ready to submit your revision, log on to https://www.editorialmanager.com/pone/ and select the 'Submissions Needing Revision' folder to locate your manuscript file.

- A letter that responds to each point raised by the academic editor and reviewer(s). You should upload this letter as a separate file labeled 'Response to Reviewers'.
- A marked-up copy of your manuscript that highlights changes made to the original version. You should upload this as a separate file labeled 'Revised Manuscript with Track Changes'.
- An unmarked version of your revised paper without tracked changes. You should upload this as a separate file labeled 'Manuscript'.

If you would like to make changes to your financial disclosure, please include your updated statement in your cover letter. Guidelines for resubmitting your figure files are available below the reviewer comments at the end of this letter.

If applicable, we recommend that you deposit your laboratory protocols in protocols.io to enhance the reproducibility of your results. Protocols.io assigns your protocol its own identifier (DOI) so that it can be cited independently in the future. For instructions see: https://journals.plos.org/plosone/s/submission-guidelines#loc-laboratory-protocols. Additionally, PLOS ONE offers an option for publishing peer-reviewed Lab Protocol articles, which describe protocols hosted on protocols.io. Read more information on sharing protocols at https://plos.org/protocols?utm_medium=editorial-email&utm_source=authorletters&utm_campaign=protocols.

As the corresponding author, your ORCID iD is verified in the submission system and will appear in the published article. PLOS supports the use of ORCID, and we encourage all coauthors to register for an ORCID iD and use it as well. Please encourage your coauthors to verify their ORCID iD within the submission system before final acceptance, as unverified ORCID iDs will not appear in the published article. *Only*  the individual author can complete the verification step; PLOS staff *cannot*  verify ORCID iDs on behalf of authors.

We look forward to receiving your revised manuscript.

Kind regards,

Bappa Mukherjee

Academic Editor

PLOS One

Journal Requirements:

When submitting your revision, we need you to address these additional requirements.

1. Please ensure that your manuscript meets PLOS ONE's style requirements, including those for file naming. The PLOS ONE style templates can be found at

https://journals.plos.org/plosone/s/file?id=wjVg/PLOSOne_formatting_sample_main_body.pdf and

https://journals.plos.org/plosone/s/file?id=ba62/PLOSOne_formatting_sample_title_authors_affiliations.pdf

2. In your Methods section, please provide additional information regarding the permits you obtained for the work. Please ensure you have included the full name of the authority that approved the field site access and, if no permits were required, a brief statement explaining why

3. Please note that PLOS One has specific guidelines on code sharing for submissions in which author-generated code underpins the findings in the manuscript. In these cases, we expect all author-generated code to be made available without restrictions upon publication of the work. Please review our guidelines at https://journals.plos.org/plosone/s/materials-and-software-sharing#loc-sharing-code and ensure that your code is shared in a way that follows best practice and facilitates reproducibility and reuse.

3. We note that the grant information you provided in the ‘Funding Information’ and ‘Financial Disclosure’ sections do not match.

When you resubmit, please ensure that you provide the correct grant numbers for the awards you received for your study in the ‘Funding Information’ section.

4.  Thank you for stating the following financial disclosure:

“The author(s) received no specific funding for this work.”

At this time, please address the following queries:

a)        Please clarify the sources of funding (financial or material support) for your study. List the grants or organizations that supported your study, including funding received from your institution.

b)        State what role the funders took in the study. If the funders had no role in your study, please state: “The funders had no role in study design, data collection and analysis, decision to publish, or preparation of the manuscript.”

c)        If any authors received a salary from any of your funders, please state which authors and which funders.

d)        If you did not receive any funding for this study, please state: “The authors received no specific funding for this work.”

Please include your amended statements within your cover letter; we will change the online submission form on your behalf.

5. Please include captions for your Supporting Information files at the end of your manuscript, and update any in-text citations to match accordingly. Please see our Supporting Information guidelines for more information: http://journals.plos.org/plosone/s/supporting-information.

6. PLOS requires an ORCID iD for the corresponding author in Editorial Manager on papers submitted after December 6th, 2016. Please ensure that you have an ORCID iD and that it is validated in Editorial Manager. To do this, go to ‘Update my Information’ (in the upper left-hand corner of the main menu), and click on the Fetch/Validate link next to the ORCID field. This will take you to the ORCID site and allow you to create a new iD or authenticate a pre-existing iD in Editorial Manager.

7. If the reviewer comments include a recommendation to cite specific previously published works, please review and evaluate these publications to determine whether they are relevant and should be cited. There is no requirement to cite these works unless the editor has indicated otherwise.

Additional Editor Comments :

Based on the reviewers comments I recommended for Major Revision.

[Note: HTML markup is below. Please do not edit.]

Reviewers' comments:

Reviewer's Responses to Questions

**Comments to the Author**

1. Is the manuscript technically sound, and do the data support the conclusions?

Reviewer #1: Yes

Reviewer #2: Yes

**********

2. Has the statistical analysis been performed appropriately and rigorously?

Reviewer #1: Yes

Reviewer #2: Yes

**********

3. Have the authors made all data underlying the findings in their manuscript fully available?

The PLOS Data policy

Reviewer #1: Yes

Reviewer #2: Yes

**********

4. Is the manuscript presented in an intelligible fashion and written in standard English?

Reviewer #1: Yes

Reviewer #2: Yes

**********

Reviewer #1: The authors have fully and effectively addressed all questions raised by the reviewers. This manuscript meets the criteria for publication, and I recommend its acceptance for publication.

Reviewer #2: I recommend acceptance after major revisions. The manuscript addresses an interesting and relevant topic and is generally well written, scientifically structured, and supported by a good-quality dataset and appropriate data analysis. The study is promising and has the potential to make a valuable contribution to the field. However, the manuscript would benefit a lot from a more in-depth discussion of the results, clearer presentation of their practical implications, and stronger integration with the existing international literature. You have to incorporate additional references to better position the findings within the current state of knowledge and to demonstrate how this study advances the field. The specific comments below are intended to strengthen the manuscript and improve its scientific impact.

Line 48: Please provide the publication year of Mandelbrot's work.

Lines 111–114: The complete USDA textural classes (e.g., silty clay loam, clay loam, sandy clay loam, silt loam, etc.) are not presented. Given the high heterogeneity of the study area, including the full textural classification would be important.

Lines 141–142: Please provide supporting references.

Lines 154–159: This section could be shortened, as it excessively describes information already presented in the table.

Line 171: The statement “which indicates a more compact soil structure at greater depths” is not fully supported, since the mean values are practically similar throughout the profile (0–240 cm).

Lines 173–174: This information is repetitive and has already been mentioned in the previous paragraph. Avoid repeating obvious descriptions of the results.

Lines 202–216: What conclusion can be drawn from this description of the results? The discussion lacks coherence and a clear interpretation of the findings.

Line 230: Please reformulate this sentence for clarity.

Lines 245–249: Where does the fractal dimension parameter appear in the model? How was matrix suction calculated? This section requires a more detailed explanation.

Line 250: The statement “air-entry values were assigned based on empirical data for different soil textures” requires clarification. How were these values assigned? This information should be explained. Table 4 presents only four values, and their origin is unclear.

Line 254: The sentence “For instance, sample RS04 exhibited the highest water retention capacity, while RS01 showed the lowest” is unnecessary. The authors should interpret the results rather than describe them so simply.

Lines 255–256: In what way are the results consistent with previous findings? How was this convergence observed? Please provide a clearer explanation.

Lines 265–269: The argumentation needs to be better supported with solid references and a more objective discussion rather than a purely qualitative interpretation.

Lines 274–275: The statement is too generic. It remains unclear how the results can be applied in practical urban management and planning.

Lines 276–281: This section is largely descriptive and somewhat repetitive of the Results section. The discussion should move beyond description and focus on interpreting the findings and addressing the underlying research problem.

Lines 286–287: What would this sampling strategy be used for? The conclusion that geological and natural factors control the spatial distribution of soil texture is rather obvious and cannot be considered a major finding of the study. What are the real implications of your results?

In the subsequent sections, particularly Section 4.3, the practical implications are discussed in greater detail. This final part of the discussion could be better organized to be more direct, less repetitive, and more focused on the study’s actual contributions.

Overall, the findings are interesting but the discussion should be more objective and should better compare the findings with studies conducted elsewhere in the world. The authors need to demonstrate more clearly how their work advances scientific knowledge and why the proposed approach is promising for practical applications.

**********

what does this mean?). If published, this will include your full peer review and any attached files.

If you choose “no”, your identity will remain anonymous but your review may still be made public.

**Do you want your identity to be public for this peer review?**  For information about this choice, including consent withdrawal, please see our Privacy Policy

Reviewer #1: No

Reviewer #2: **Yes:** Thomas Vincent Gloaguen

**********

[NOTE: If reviewer comments were submitted as an attachment file, they will be attached to this email and accessible via the submission site. Please log into your account, locate the manuscript record, and check for the action link "View Attachments". If this link does not appear, there are no attachment files.]

To ensure your figures meet our technical requirements, please review our figure guidelines: https://journals.plos.org/plosone/s/figures

You may also use PLOS’s free figure tool, NAAS, to help you prepare publication quality figures: https://journals.plos.org/plosone/s/figures#loc-tools-for-figure-preparation.

NAAS will assess whether your figures meet our technical requirements by comparing each figure against our figure specifications.

# Author comment: Author response to Decision Letter 1

**DOI:** 10.1371/journal.pone.0357100.r003

13 Jul 2026

We thank the editors and two anonymous reviewers for their valuable and constructive comments, which have greatly helped improve the quality of our manuscript. Every critical suggestion has been carefully reviewed and fully addressed in the revision. We have systematically revised the entire manuscript, and specifically added more in-depth discussion and extended the analysis of the research implications in the Discussion section as suggested. All revisions are marked with red track changes for easy identification. Detailed point-by-point responses to the comments from Reviewer #1 and Reviewer #2 are presented below.

Response to Reviewer 1#:

Comments.

The authors have fully and effectively addressed all questions raised by the reviewers. This manuscript meets the criteria for publication, and I recommend its acceptance for publication.

Author response:

We appreciate your positive evaluation of our revised manuscript.

Response to Reviewer 2#:

Comment 1.

I recommend acceptance after major revisions. The manuscript addresses an interesting and relevant topic and is generally well written, scientifically structured, and supported by a good-quality dataset and appropriate data analysis. The study is promising and has the potential to make a valuable contribution to the field. However, the manuscript would benefit a lot from a more in-depth discussion of the results, clearer presentation of their practical implications, and stronger integration with the existing international literature. You have to incorporate additional references to better position the findings within the current state of knowledge and to demonstrate how this study advances the field. The specific comments below are intended to strengthen the manuscript and improve its scientific impact.

Author response:

Thank you very much for your valuable and constructive comments. We have revised the manuscript as you suggested.

Comment 2.

Line 48: Please provide the publication year of Mandelbrot's work.

Author response:

We have clearly marked the publication year 1977 in reference [15] for Mandelbrot’s work. The complete citation is: [15] Mandelbrot BB. Fractals: from, chance, and dimension. San Francisco: WH Freeman; 1977.

Comment 3.

Lines 111–114: The complete USDA textural classes (e.g., silty clay loam, clay loam, sandy clay loam, silt loam, etc.) are not presented. Given the high heterogeneity of the study area, including the full textural classification would be important.

Author response:

This study is oriented toward the practical engineering application of sponge city construction. The particle size classification adopted follows the Standard for Engineering Classification of Soil (Chinese national standard), which is universally used in domestic hydrogeological surveys and municipal engineering design. This classification system is directly compatible with the technical specifications of local sponge city construction, ensuring that our research conclusions can be directly applied to engineering practice. According to Standard for Engineering Classification of Soil, the dominant soil textures classes are clarified silt, silty clay and clay in the study area. The revised content has been updated in the manuscript. (Lines 125-128, new MS)

Comment 4.

Lines 141–142: Please provide supporting references.

Author response:

We have added supporting references [27,28] at lines 155–156 (lines 141–142, old MS) in the revised manuscript. The complete citations are: [27] Wang JM, Lu X, Feng Y, Yang RX. Integrating multi-fractal theory and geo-statistics method to characterize the spatial variability of particle size distribution of minesoils. Geoderma, 2018; 317: 39-46. [28] Wang JM, Zhang JR, Feng Y. Characterizing the spatial variability of soil particle size distribution in an underground coal mining area: An approach combining multi-fractal theory and geostatistics. Catena 2019; 176, 94-103.

Comment 5.

Lines 154–159: This section could be shortened, as it excessively describes information already presented in the table.

Author response:

We have shortened this section and removed redundant details already presented in the table. (Lines 168-171, new MS)

Comment 6.

Line 171: The statement “which indicates a more compact soil structure at greater depths” is not fully supported, since the mean values are practically similar throughout the profile (0–240 cm).

Author response:

We have revised this statement. The original inference about compact soil structure was not sufficiently supported by the data. The revised text only describes the narrower variation range and higher homogeneity of D values at greater depths, without overinterpreting soil structural characteristics. (Lines 182-183, new MS)

Comment 7.

Lines 173–174: This information is repetitive and has already been mentioned in the previous paragraph. Avoid repeating obvious descriptions of the results.

Author response:

We have shortened this section and removed the repetitive descriptions already stated in the previous paragraph. (Lines 184-185, new MS)

Comment 8.

Lines 202–216: What conclusion can be drawn from this description of the results? The discussion lacks coherence and a clear interpretation of the findings.

Author response:

We have revised this section. We added a clear concluding interpretation of the spatial distribution pattern, sorted out the logical link between D value characteristics, formation causes and soil particle composition, and improved the coherence of result description and interpretation. (Lines 213-229, new MS)

Comment 9.

Line 230: Please reformulate this sentence for clarity.

Author response:

We have reformulated this sentence. We corrected the grammatical error and clarified the logical relationship between the influencing factors and the depth-dependent uniformity of D values. (Line 243-246, new MS)

Comment 10&11.

Lines 245–249: Where does the fractal dimension parameter appear in the model? How was matrix suction calculated? This section requires a more detailed explanation.

Line 250: The statement “air-entry values were assigned based on empirical data for different soil textures” requires clarification. How were these values assigned? This information should be explained. Table 4 presents only four values, and their origin is unclear.

Author response:

We appreciate the reviewer’s insightful question. The fractal dimension parameter appears in the model through its empirical and theoretical linkage to the van Genuchten (VG) soil water characteristic curve (SWCC) parameters. As detailed in the new revised manuscript, we employed the Bird et al. (2000) and Huang et al. (2002) fractal model related D to predict the SWCCs. (Lines 256-270)

We have clarified the assignment rule and origin of the air-entry values. We explained in the text that the values are matched to soil texture classes and sourced from a classic empirical dataset, with a supporting reference [37] added. (Lines 273-280)

Comment 12&13.

Line 254: The sentence “For instance, sample RS04 exhibited the highest water retention capacity, while RS01 showed the lowest” is unnecessary. The authors should interpret the results rather than describe them so simply.

Lines 255–256: In what way are the results consistent with previous findings? How was this convergence observed? Please provide a clearer explanation.

Author response:

We have revised this section to address both comments.

For Line 254: We removed the simple descriptive statement of individual samples, and replaced it with an interpretive explanation of the pore-scale mechanism linking fractal dimension to water retention capacity. (Lines 283-289, new MS)

For Lines 255–256: We clarified that the consistency with previous findings lies in the positive correlation between fractal dimension and water retention performance, which matches the established conclusion in fractal soil hydrology research. Corresponding supporting references [11,38] have been added. (Lines 287-287, new MS)

Comment 14.

Lines 265–269: The argumentation needs to be better supported with solid references and a more objective discussion rather than a purely qualitative interpretation.

Author response:

We have revised this section. We supplemented additional references to support the relationship between soil texture and hydraulic properties, linked the engineering implication to the study area’s climatic characteristics with regional research evidence, and added notes on other influencing factors to make the discussion more objective and evidence-based. (Lines 297-307, new MS)

Comment 15.

Lines 274–275: The statement is too generic. It remains unclear how the results can be applied in practical urban management and planning.

Author response:

We have revised this generic statement. We clarified the specific practical application paths of the results, including hydrological functional zoning, targeted sponge facility layout, and optimization of geological survey workload in urban planning and management, to make the practical application value of the findings more explicit. (Lines 313-322, new MS)

Comment 16.

Lines 276–281: This section is largely descriptive and somewhat repetitive of the Results section. The discussion should move beyond description and focus on interpreting the findings and addressing the underlying research problem.

Author response:

We have revised this section. We removed the descriptive content that repeated the Results section, and shifted the focus to interpretation: we explained the genetic mechanism behind the vertical differentiation of fractal dimensions, clarified the advantage of fractal dimension as a comprehensive indicator over traditional grain-size parameters, and discussed the practical value of this finding for optimizing hydrological model parameterization and improving simulation accuracy in sponge city planning. (Lines 323-327, new MS)

Comment 17.

Lines 286–287: What would this sampling strategy be used for? The conclusion that geological and natural factors control the spatial distribution of soil texture is rather obvious and cannot be considered a major finding of the study. What are the real implications of your results?

Author response:

We have reorganized this section of the discussion. We removed the specific descriptions of sponge facility layout that overlap with Section 4.3, and shifted the focus to the core contribution of this study: the fractal-based method provides an efficient solution for obtaining deep soil hydraulic parameters at the regional scale, addressing a common limitation of traditional measurement methods. The revised content is more concise, avoids repetition with subsequent sections, and logically leads to the detailed practical implications in Section 4.3. (Lines 331-337, new MS)

Comment 18.

In the subsequent sections, particularly Section 4.3, the practical implications are discussed in greater detail. This final part of the discussion could be better organized to be more direct, less repetitive, and more focused on the study’s actual contributions.

Author response:

We sincerely appreciate the reviewer’s constructive suggestion regarding the practical implications in Section 4.3. We agree that the previous discussion was somewhat diluted by redundant background information and lacked a sharp focus on actionable outcomes. To address this, we have thoroughly revised the section (titled “Optimization of Sponge City Facility Design Based on Fractal Characteristics” in the new MS) by removing repetitive descriptions of general sponge city principles and reorienting the narrative toward specific, data-driven insights. The revised content explicitly translates our findings into engineering guidelines—for instance, specifying that soil fractal dimensions (D) between 2.60 and 2.75 require 15–20 cm crushed stone layers to prevent clogging, and that depression storage depths of 30–50 cm optimize retention in high-D zones. Furthermore, we have strengthened the discussion on spatial decision-making by mapping these quantitative thresholds to local contexts, such as recommending permeable pavements for the Xingfu and Zhujiace communities (D<2.65) and engineered rain gardens for the Fu River alluvial plains (D>2.75). These refinements ensure the discussion is concise, targeted, and provides a robust, quantitative foundation for optimizing sponge city designs in analogous hydrogeological settings. (Line 358-420, new MS)

Comment 19.

Overall, the findings are interesting but the discussion should be more objective and should better compare the findings with studies conducted elsewhere in the world. The authors need to demonstrate more clearly how their work advances scientific knowledge and why the proposed approach is promising for practical applications.

Author response:

We have revised the discussion section. We added comparisons with international studies to place our findings in the global research context, and clarified that this study extends fractal analysis from shallow topsoil to deep urban vadose zones, advancing the application scope of existing methods. We also explained the practical advantages of the fractal-based approach in survey efficiency and cost, as well as its generalizability to similar regions. The revised discussion is more objective, with more explicit scientific contributions and application prospects. (Lines 357-420, new MS)

Attachment Submitted filename: Response to Reviewers-PONE-D-26-14596.docx

# Peer review: Decision Letter 1

**DOI:** 10.1371/journal.pone.0357100.r004

**Authors:** Mohamed Hanfi

12 Aug 2026

Spatial variability of soil texture fractal dimension and its application in sponge city construction

PONE-D-26-14596R1

Dear Dr. Yun Li,

We’re pleased to inform you that your manuscript has been judged scientifically suitable for publication and will be formally accepted for publication once it meets all outstanding technical requirements.

Within one week, you’ll receive an e-mail detailing the required amendments. When these have been addressed, you’ll receive a formal acceptance letter and your manuscript will be scheduled for publication.

An invoice will be generated when your article is formally accepted. Please note, if your institution has a publishing partnership with PLOS and your article meets the relevant criteria, all or part of your publication costs will be covered. Please make sure your user information is up-to-date by logging into Editorial Manager at Editorial Manager® and clicking the ‘Update My Information' link at the top of the page. For questions related to billing, please contact billing support.

If your institution or institutions have a press office, please notify them about your upcoming paper to help maximize its impact. If they’ll be preparing press materials, please inform our press team as soon as possible -- no later than 48 hours after receiving the formal acceptance. Your manuscript will remain under strict press embargo until 2 pm Eastern Time on the date of publication. For more information, please contact onepress@plos.org.

Kind regards,

Mohamed Y.M. Hanfi

Academic Editor

PLOS One

Additional Editor Comments (optional):

Reviewers' comments:

Reviewer's Responses to Questions

**Comments to the Author**

Reviewer #1: All comments have been addressed

Reviewer #2: All comments have been addressed

**********

2. Is the manuscript technically sound, and do the data support the conclusions?

Reviewer #1: Yes

Reviewer #2: Yes

**********

3. Has the statistical analysis been performed appropriately and rigorously?

Reviewer #1: Yes

Reviewer #2: Yes

**********

4. Have the authors made all data underlying the findings in their manuscript fully available?

The PLOS Data policy

Reviewer #1: Yes

Reviewer #2: Yes

**********

5. Is the manuscript presented in an intelligible fashion and written in standard English?

Reviewer #1: Yes

Reviewer #2: Yes

**********

Reviewer #1: All comments from the previous revision have been thoroughly and effectively addressed. I recommend acceptance of the manuscript, pending a response to the following minor point:

As shown in Table 3, the Spherical model yields a determination coefficient (R²) of only 0.10 for the 200–220 cm depth layer and only 0.37 for the 220–240 cm layer (Gaussian model), reflecting extremely poor goodness of fit. This finding indicates that the spatial structure of soil fractal dimension is very weak at these depths, and the resulting interpolation outputs have limited reliability. The authors are requested to discuss the potential underlying causes of this pattern in the Discussion section.

Reviewer #2: The authors have answered all questions and satisfactorily revised the manuscript. My recommendation now is acceptance.

**********

what does this mean?). If published, this will include your full peer review and any attached files.

If you choose “no”, your identity will remain anonymous but your review may still be made public.

**Do you want your identity to be public for this peer review?**  For information about this choice, including consent withdrawal, please see our Privacy Policy

Reviewer #1: No

Reviewer #2: **Yes:** Thomas Vincent Gloaguen

**********

# Editor report: Acceptance letter

**DOI:** 10.1371/journal.pone.0357100.r005

**Authors:** Mohamed Hanfi

PONE-D-26-14596R1

PLOS One

Dear Dr. Li,

I'm pleased to inform you that your manuscript has been deemed suitable for publication in PLOS One. Congratulations! Your manuscript is now being handed over to our production team.

At this stage, our production department will prepare your paper for publication. This includes ensuring the following:

* All references, tables, and figures are properly cited

* All relevant supporting information is included in the manuscript submission,

* There are no issues that prevent the paper from being properly typeset

You will receive further instructions from the production team, including instructions on how to review your proof when it is ready. Please keep in mind that we are working through a large volume of accepted articles, so please give us a few days to review your paper and let you know the next and final steps.

Lastly, if your institution or institutions have a press office, please let them know about your upcoming paper now to help maximize its impact. If they'll be preparing press materials, please inform our press team within the next 48 hours. Your manuscript will remain under strict press embargo until 2 pm Eastern Time on the date of publication. For more information, please contact onepress@plos.org.

You will receive an invoice from PLOS for your publication fee after your manuscript has reached the completed accept phase. If you receive an email requesting payment before acceptance or for any other service, this may be a phishing scheme. Learn how to identify phishing emails and protect your accounts at https://explore.plos.org/phishing.

If we can help with anything else, please email us at customercare@plos.org.

Thank you for submitting your work to PLOS One and supporting open access.

Kind regards,

PLOS One Editorial Office Staff

on behalf of

Dr. Mohamed Y.M. Hanfi

Academic Editor

PLOS One
